# Novel Psychoactive Substances—Recent Progress on Neuropharmacological Mechanisms of Action for Selected Drugs

**DOI:** 10.3389/fpsyt.2017.00152

**Published:** 2017-08-18

**Authors:** Zurina Hassan, Oliver G. Bosch, Darshan Singh, Suresh Narayanan, B. Vicknasingam Kasinather, Erich Seifritz, Johannes Kornhuber, Boris B. Quednow, Christian P. Müller

**Affiliations:** ^1^Centre for Drug Research, Universiti Sains Malaysia, Minden, Malaysia; ^2^Department of Psychiatry, Psychotherapy and Psychosomatics, Psychiatric Hospital, University of Zurich, Zurich, Switzerland; ^3^School of Social Sciences, Universiti Sains Malaysia, Minden, Malaysia; ^4^Department of Psychiatry and Psychotherapy, University Hospital, Friedrich-Alexander University Erlangen-Nuremberg, Erlangen, Germany; ^5^Experimental and Clinical Pharmacopsychology, Department of Psychiatry, Psychotherapy and Psychosomatics, Psychiatric Hospital, University of Zurich, Zurich, Switzerland

**Keywords:** Kratom, synthetic cannabinoids, dimethyltryptamine, serotonergic hallucinogens, mephedrone, ketamine, γ-hydroxybutyrate

## Abstract

A feature of human culture is that we can learn to consume chemical compounds, derived from natural plants or synthetic fabrication, for their psychoactive effects. These drugs change the mental state and/or the behavioral performance of an individual and can be instrumentalized for various purposes. After the emergence of a novel psychoactive substance (NPS) and a period of experimental consumption, personal and medical benefits and harm potential of the NPS can be estimated on evidence base. This may lead to a legal classification of the NPS, which may range from limited medical use, controlled availability up to a complete ban of the drug form publically accepted use. With these measures, however, a drug does not disappear, but frequently continues to be used, which eventually allows an even better estimate of the drug’s properties. Thus, only in rare cases, there is a final verdict that is no more questioned. Instead, the view on a drug can change from tolerable to harmful but may also involve the new establishment of a desired medical application to a previously harmful drug. Here, we provide a summary review on a number of NPS for which the neuropharmacological evaluation has made important progress in recent years. They include mitragynine (“Kratom”), synthetic cannabinoids (e.g., “Spice”), dimethyltryptamine and novel serotonergic hallucinogens, the cathinones mephedrone and methylone, ketamine and novel dissociative drugs, γ-hydroxybutyrate, γ-butyrolactone, and 1,4-butanediol. This review shows not only emerging harm potentials but also some potential medical applications.

## Introduction

It appears to be a human trait to constantly seek for new psychoactive substances and to explore potential use of them. As long as human record keeping dates back, humans consume psychoactive plant preparations. Since centuries they isolated single compounds yielding “natural drugs,” while since decades synthetic chemistry allowed the innovation of completely new compounds that are not available from natural resources (1, 2). Despite the risk of being toxic upon single or chronic consumption, there are constantly new drugs that find their way into drug-taking communities (3).

Novel stimuli and novel information about the external world have an incentive salience and maintain seeking behavior in animals and in humans (4, 5). The search for novel external stimuli may translate to novel “mental states,” as an experience of new interoceptive states. Human brains generate distinct working modes that are subjectively perceived as mental states. This is at the neurobiological side believed to be organized by the summatory tonic activity of modulatory transmitter systems. Mental states can determine how an organism perceives, processes, and stores external and internal information. It also affects how efficiently behavior is generated (6–8). Mental states change spontaneously or as a consequence of environmental influences, thereby some mental states are perceived as more pleasurable and useful for goal-directed behavior than others. The rewarding value of novelty may, thus, be expanded to novel mental states, which have never been incurred by natural means. Psychoactive substances can induce and maintain a desired mental state. Some of them may also provoke novel mental states. Only a few of the well-established psychoactive substances induce “euphoria” or a sense of “well-being,” which directly reinforces drug-seeking and consumption behaviors. Most psychoactive substances, however, induce mental states that are primarily useful for other purposes. In that, they exert complex reinforcing effects during drug instrumentalization (6–10). Thus, the mental state that is induced by a psychoactive drug and for which humans develop a memory (11) may facilitate other behaviors with positive or negative reinforcing outcome, such as the facilitation of social interaction, mating behavior, coping with stress, and cognitive enhancement (9, 12–16). When a new drug is discovered and experimentally used, the new user may not only judge the novelty and emotional impact of the newly experienced mental state but subsequently decide for what purposes this new mental state may serve (17, 18). Once a new drug is made available, an experimental consumption starts that determines individual subjective effects as well as context and possibility of instrumentalization. This may not only work in humans but also for a newly experienced psychoactive drug in animals (19).

A major factor that fuels continuous search for new psychoactive drugs is the need to replace existing ones in routine use. Once a long known drug has been criminalized and banned, availability of the drug becomes limited. Legal control imposes punishment on drug possession, trade, and use, which limits its instrumentalization for frequently performed behavior, such as coping with stress (20). If the drug was useful for this behavior, e.g., to better relax after stressful work, users may start looking for a legal replacement of the banned drug and, thus, be motivated for testing new drugs (21).

Novel psychoactive substances (NPSs) had been defined by the United Nations as new narcotic or psychotropic drugs that are not controlled by the United Nations’ 1961 Single Convention on Narcotic Drugs or by Psychotropic Substances Conventions (22). NPSs are by definition those psychoactive drugs used for intoxication which are not already prohibited by UN Single Convention on Narcotic Drugs or Misuse of Drugs Act (23), thereby “novel” does not necessarily mean that the drug has been developed completely new recently. It may also refer to substances that have lately become popular and/or more widely available, constituting a reason of current or potential public health concerns (24).

The way of a NPS in society, from its introduction, experimental use, instrumentalization, habitual abuse, up to its legal control, depends essentially on the relationship of adverse side effects and potential medical use. The adverse side effects are those effects of the drug that threaten the physiological integrity and behavioral repertoire of the whole organism, beyond the desired psychoactive action. Many known psychoactive drugs are strong toxins and harm the user. This can occur after acute consumption or after chronic intake (3). Humans establish cultural rules for the consumption and the control of side effects of psychoactive drugs (25). This keeps even highly dangerous drugs, such as alcohol, legal and limits their harm potential when incorporated in cultural activities (26). But establishing those initially “non-written” rules requires a certain amount of experience and a user/non-user discourse. One result of this discourse is the possibility of legal control, and a “written down” law on where, when, and how a psychoactive substance can be used. Drugs can be labeled as addictive drugs and made illicit. However, many new substances are at the same time tested for a potential beneficial application, e.g., to treat pain, or even as substitutes for well-known addictive drugs, thereby the verdict may be that a NPS might have some addiction- and harm potential but also beneficial effects, which may actually dominate the use profile. There is occasionally also a reversal of the discourse decision in that addictive drugs may receive an additional medical application, e.g., ketamine, which was discussed as an abuse drug (27, 28), but is now also considered as a useful treatment for depression (29).

Newly introduced psychoactive substances do not usually arise from controlled pharmacodevelopment. In that, these drugs initially have the status of a “legal high” and virtually everybody is allowed to possess, distribute, and consume them. Only when after consumption accidents with physical- and/or behavioral impairments occur, or in the worst case drug fatalities, a NPS can be classified and legally controlled or its medical use defined (21). However, the drug discourse requires evidence, ideally scientific, arising from controlled experimentation. This evidence should go well beyond the accumulation of single cases. Quite naturally, during the information collecting period, the NPS is used, and thus, not brand new anymore. What is new afterward is the certainty with which sufficiently reliable statements about the drug can be made (30).

It has to be admitted that the legal status discourse is in practice way more complicated and also culturally selective, which shall not be the focus of this review that rather focuses on the neurobiological evidence that feeds into this discourse. In this article, we review the state of knowledge on a number of NPS for which now a considerable penetration of society has developed in distinct cultural or geographical regions and for which sufficient evidence has been gathered to allow for evidence-based statements. This should provide a comprehensive overview on some of the currently most relevant NPS, thereby the choice of substances discussed was driven by the perceived progress in the understanding of their neuropharmacological action by the authors. In that, the review does not provide a complete coverage of all currently available NPS.

## Kratom and Mitragynine

*Mitragyna speciosa Korth*. (*M. speciosa*), from the Rubiaceae family, is a tropical medicinal plant native to Southeast Asia (31, 32). In Malaysia, *M. speciosa* leaves are known as Ketum or Biak (31, 32), and in Thailand as Kratom (33, 34). *M. speciosa* has been historically used in Southeast Asia as a stimulant drug and in its traditional context as a remedy for various symptoms (33, 34). Previous studies mainly described the traditional uses of Kratom among rural folk, peasants, and laborers in Southeast Asia (33, 35, 36). More recently, studies on Kratom use are emerging from Europe and the US (37–40). They suggest that Kratom is now also used outside its traditional context. In the West, it is still considered a “safe” herbal drug with potential medicinal application (38, 39, 41, 42).

In Southeast Asia, manual laborers commonly chew fresh Kratom leaves and ingest brewed Kratom tea/juice to reduce fatigue, promote work desire, and enhance physical tolerance to debilitating work (32, 33, 43, 44). Kratom leaves are also used as an opium substitute to treat morphine addiction in Malaysia and Thailand (31, 45). Because of its unique healing properties, rural inhabitants use Kratom leaves to treat various medical conditions such as cough, fever, pain, diarrhea, diabetes, and hypertension (32, 44, 46). However, Kratom is potentially addictive and chronic users find it difficult to refrain from prolonged Kratom use (33, 36, 43). The common side effects of long-term use include constipation, weight loss, hyperpigmentation, dehydration, fatigue, insomnia, and increased urination (33, 36, 46). The majority of Kratom users believe its use is not as harmful as those of other illicit substances, such as methamphetamine and heroin, and that Kratom dependence carries little or no health risks (45–47). So far, there have been no deleterious incidents directly related to Kratom use in Southeast Asia. Only one study from Thailand has reported Kratom poisoning cases, with palpitation, seizure, and nausea. However, these effects may have been arisen from coadministration of other illicit substances (48).

Regular users are more likely to increase the quantity and frequency of Kratom use over time. In Thailand, traditional users often chew fresh or powdered Kratom leaves (33, 44). In Malaysia, Kratom users commonly ingest brewed Kratom tea/juice (25, 36, 47, 49). In the US and in Europe, Kratom is primarily used as a natural alternative to self-medicate for chronic pain and as an opioid withdrawal treatment (37, 50, 51). Kratom is marketed as a “legal high” and can be easily obtained in different forms, such as powder extracts, tablets, capsule, or liquids, through the Internet (38, 52). As a consequence of the rise in Kratom mortality and toxicity cases in the West, regulatory agencies have begun to view Kratom negatively (39, 53, 54). The US Drug Enforcement Administration intends to regulate Kratom use in the US (51). However, it appears that most, if not all of the Kratom-induced medical complications in the West were triggered by the use of adulterated Kratom products (53, 54).

About 40 alkaloids have been identified in *M. speciosa* leaves. The alkaloid content in the leaves varies, depending on geographical location and season of harvest (55). Mitragynine and 7-hydroxymitragynine are the principal psychoactive constituents of *M. speciosa* and were shown to induce morphine-like effects in animal models (31, 55, 56). The synthesis of the mitragynine was reported by Takayama et al. (57) and later by Ma and colleagues (58, 59). However, a synthesis of mitragynine is with 18–23 steps rather laborious, time-consuming, not economical, and has only a low yield of 3–13% (60). Thus, direct isolation of mitragynine from the leaves is much more efficient and cost-effective.

A comprehensive pharmacokinetic description of mitragynine in rats was provided by Parthasarathy et al. (61) after intravenous (i.v.) and oral administration. The blood concentration peaked at 1.2 h with 2.3 µg/mL followed by biphasic elimination with a half-life of 2.9 h and a clearance of 0.09 L/h/kg after administration of 1.5 mg/kg mitragynine (i.v.). The volume of distribution was rather small with 0.79 L/kg, suggesting that mitragynine is not widely distributed into tissue compartments (62). The oral absorption of mitragynine was shown to be lengthy and incomplete, with an absolute oral bioavailability of around 3%. Several studies revealed that after oral application of 20–50 mg/kg mitragynine, a volume distribution of 37–89 L/kg and clearance of 1.6–7 L/h (per kg) was reached (61–63), which supports the low bioavailability and poor absorption of mitragynine.

Mitragynine is a lipophilic alkaloid with poor water solubility (64). Mitragynine has a biphasic metabolism. The first phase produces seven identified metabolites, thereby mitragynine is processed through hydrolysis of methyl ester in position 16 and O-demethylation of the 9-methoxy- and of the 17-methoxy groups (65). The second phase involves further oxidation to carboxylic acids or reduction to alcohols and the combinations of some steps *via* the intermediate aldehydes. Four metabolites were additionally conjugated to glucuronides and to sulfates in rats and humans (65). Abuse of mitragynine and related compounds can be detected through gas chromatography or liquid chromatography with mass spectrometry, respectively (65–67).

Mitragynine shows the highest affinity to κ-opioid receptors followed by μ- and δ-opioid receptors (68). It acts as a receptor agonist at μ-opioid receptors and possibly as an antagonist at κ-opioid receptors (56, 69–71). At cellular level, mitragynine blocks neuronal Ca^2+^ channels (72). It was also found to inhibit forskolin-stimulated cyclic adenosine monophosphate (cAMP) formation *in vitro* in an opiate receptor-dependent way (73, 74). A study by Fakurazi et al. (75) showed that repeated exposure to mitragynine attenuated the expression of cAMP and cAMP response element-binding protein.

Mitragynine was extensively investigated for its antinociceptive effects. A study by Reanmongkol et al. (76) found prolonged antinociceptive effects in the hot plate test, but not in the tail flick test. Another study showed prolonged antinociceptive effect in both tests (77). Intraperitoneal administration also yielded positive antinociceptive results in the hot plate, formalin-, and acetic-acid tests (78). Mitragynine’s antinociceptive effects were comparable to those of oxycodone suggesting an abuse potential (79, 80).

In animal models, mitragynine induces anxiolytic effects after acute treatment in several test paradigms (81). This may be mediated by its effects on Fos expression in dorsal raphe nucleus (82), and the activation of δ-opioid receptors (83). Withdrawal from chronic mitragynine induces anxiety-related behavior in rats (84). There have been conflicting reports of mitragynine affecting cognitive function. Apryani et al. (85) found that mitragynine i.p. administration can impair object location memory in mice. Another study, however, showed no impairment of short-term memory in the Y-maze task. The mice, however, were given *M. speciosa* extract through oral administration (86). In rats, a study showed an increase in learning ability when given *M. speciosa* extract in a passive- and an active avoidance task. However, mitragynine alone did not have significant effects on long-term memory consolidation in both tasks (87). A recent study using a passive avoidance task showed independent impairments of learning, memory consolidation, as well as memory retrieval after acute mitragynine administration at a dose ≥10 mg/kg (i.p.) in rats. In parallel, mitragynine-treated rats showed a disrupted low frequency rhythm (delta and theta) in the electroencephalogram (EEG), which may account for the learning and memory impairments (84).

Chronic administration of mitragynine at a dose of ≥10 mg/kg (i.p.) may cause addiction-like behaviors in animal models (56, 84, 88). Mitragynine (15 mg/kg, i.p.) shows discriminative stimulus properties in rats. It fully substituted for a morphine (5 mg/kg) stimulus, and partially for a cocaine cue (10 mg/kg, i.p.) (89). Thus, mitragynine likely possess both opioid and psychostimulant effects. Mitragynine at doses ≥10 mg/kg (i.p.) shows rewarding properties in rodents as measured by conditioned place preference. These effects are opiate receptor dependent and can be blocked by the opiate receptor antagonist naloxone (56). Subchronic administration of mitragynine increased the expression of dopamine transporter- and dopamine (DA) receptor-regulating factor mRNA in the limbic system of the brain (84) indicating a critical role of DA in the rewarding effects of mitragynine, thereby the dose of mitragynine may be crucial, given that addiction-like behaviors were only observed at doses ≥10 mg/kg (i.p.) in rodents. Those are considerably higher than reported maximum doses of mitragynine consumed by humans, which are usually in the range of <3 mg/kg (p.o.) per day.

Altogether, Kratom and its main psychoactive ingredient mitragynine are drugs that are widely used in Southeast Asia with an increasing appearance in Western countries. Experimental studies in animals have now shown that mitragynine has an addictive potential, however, only at higher doses. Human users in countries of frequent use with a traditional context report a rather low daily consumption with only mild side effects. Kratom and mitragynine can be instrumentalized to enhance physical work power and endurance. A major reason for Kratom consumption is its reported efficacy to replace opiates in chronic users. This makes the Kratom plant preparation and also the isolated compound mitragynine interesting options to treat opiate addiction.

## Synthetic Cannabinoids

The abuse of herbal preparations spiked with synthetic cannabinoids is still increasing. A hallmark of this consumption is the use of an inhomogeneous group of substances that occur on the market with different names, such as Spice, Spice gold, diamond-spice, chill X, abyss, Pandora’s box, exodus, annihilation, fire, smoke, sence, chillX, chillys, highdi’s, earth impact, and many more (90, 91). Synthetic cannabinoid preparations are frequently mislabeled as research chemicals, herbal incenses, or as legal highs, including the explicit warning that it is not for human consumption (92–97). The first evidence of synthetic cannabinoid use as a recreational drug appeared in 2004 (98). However, a wide spread use of synthetic cannabinoids did not emerge until 2008. In 2012, the lifetime prevalence for “Spice” consumption was already at 7% among the 15- to 18-year olds (99–101), thereby the coabuse of synthetic- and natural cannabinoids is common (102–109).

Research in the active ingredients of synthetic cannabinoids such as Spice and their neuropharmacological action has revealed several hundred compounds that are artificially added to a carrier medium of herbal origin (110). The synthetic compounds usually display a high affinity for cannabinoid receptors (CB-R), which reaches far beyond that of natural cannabinoids (100, 111). Compared to the partial agonist, Δ9-tetrahydrocannabinol (THC), synthetic cannabinoids can act as agonists, neutral antagonists, or inverse agonists at the CB-R1 (110–112). Synthetic cannabinoid preparations also lack the naturally occurring cannabidiol, which is present in cannabis preparations and which is supposed to antagonize some of the psychotogenic effects of THC (113, 114).

A gram of herbal preparation can contain up to 200 mg of a synthetic cannabinoid. However, the variability in substance composition and amount between one package and another is high and largely unpredictable. Additional ingredients have been found and may include, e.g., clenbuterol, which may be responsible for the frequently observed sympathomimetic manifestations of an intoxication with synthetic cannabinoids, or tocopherol. The latter is usually added to blur chemical detection (113–115). Occasionally, some investigated herbal preparations did not contain any pharmacologically active synthetic cannabinoids, but only psychoactive compounds from plant-derived carrier material, such as mitragynine (116–120).

Users report that synthetic cannabinoids can cause psychotropic effects that are qualitatively similar, but much more intense, than those of cannabis. As such, synthetic cannabinoids may cause THC-like effects including alterations of mood, sleep, perception/wakefulness, body temperature, and cardiovascular function (121–123). Additional diffuse effects, which are different from cannabis, include palpitations, tachycardia, and unspecific effects in the electrocardiogram (110, 124, 125). Harmful somatic effects comprise gastrointestinal and renal defects (91, 126–128). Neuropsychiatric symptoms were reported, such as psychosis, panic and anxiety attacks, agitation, and aggressive behavior (106, 107, 129, 130). A psychosis induced by synthetic cannabinoids manifests by delusions, acoustic and visual hallucinations, and paranoia. Neurological symptoms may include seizures, dystonia, and tremors. Other frequently reported side effects are nausea, vomiting, diaphoresis, and respiratory depression (131–141). Use of synthetic cannabinoids may have fatal consequences. Reported single cases mention coronary ischemic events and suicide caused by an extreme anxiety attack (138, 139).

The active compound of the preparation “Spice” was first described in 2009, following the detection of formerly non-declared, synthetic CB-R1 agonists (141, 142). Synthetic cannabinoids were originally developed for research purposes in the 1970s with the goal of better understanding the endogenous cannabinoid pathways and to develop pharmacotherapies for conditions such as cancer-associated pain (108). Synthetic cannabinoids may contain aminoalkyl-indoles of the JWH series, which was first synthesized by the chemist J. W. Huffmann. Major ingredients of herbal preparations in the past included the aminoalkyl-indoles, JWH-018, JWH-073, JWH-019, JWH-250, and the cyclohexylphenols, CP-47,497-C6, CP-47,497, and CP-47,497-C8. These compounds are lipid-soluble, non-polar, and typically contain 20–26 carbon atoms. However, there are at least 100 chemically related compounds currently known (122, 143–146). While some of them have been legally controlled on individual level, recent legislation in Germany now considers the lead structures and attempts to control whole drug classes. It is expected that this will make it more difficult to simply replace single banned compounds by their substituted analogs in the synthetic cannabinoid preparations (122, 147–153).

At the current stage, one may conclude that synthetic cannabinoids constitute dangerous psychoactive drug preparations with a rather chimeric nature (154). It is not a single compound, but draws from a plethora of already available synthetic cannabinoids that are unsystematically mixed and brought on a plant carrier material, that may even by itself have psychoactive effects. This strategy of drug preparation paved the way into the perception as a natural and perfectly “legal high” by consumers. The natural claim is now clearly rejected by the understanding that most psychoactive effects are brought about by purely synthetic compounds added to a natural carrier. Given the strong cannabinoid-like effects of synthetic cannabinoids, which are now increasingly understood, single substances have been legally banned. But this has done little damage to the unique drug design of synthetic cannabinoid preparations in that single disallowed compounds were almost immediately replaced by substituted analogs that had not been banned yet. The now emerging control of whole substance classes will most likely put an end to this strategy and help to reduce harm that is clearly associated with synthetic cannabinoid consumption.

## Dimethyltryptamine

*N*-*N*-dimethyltryptamine (DMT) is an indole alkaloid found in plants and animals. It has been proposed that the endogenous DMT may act as a neurotransmitter. DMT is a natural psychedelic substance and has similar effects as other serotonergic hallucinogens such as lysergic acid diethylamide (LSD), psilocybin, and mescaline. DMT is one of the ingredients used in various shamanic preparations, such as ayahuasca, hoasca, or yagé in South America and is used as a recreational drug in Europe and North America (155). DMT rich plants belong to genera such as *Phalaris, Delosperma, Acacia, Desmodium, Mimosa, Virola*, and *Psychotria*. When DMT is ingested at high concentrations, the user experiences episodic visual hallucinations (155, 156). The recreational use of DMT has been rising for its acclaimed self-perceived benefits. Capsules, known as pharmahuasca, became available containing DMT as a free base together with some monoamine oxidase inhibitors (MAOIs), such as synthetic harmaline, or plant-based MAOIs such as Harmala alkaloids (157, 158). The MAOIs inhibit the otherwise rapid metabolization of DMT and, thus, allow for the hallucinogenic effects when the drug is taken orally.

Endogenous DMT can be found in the human brain and other tissues of the body such as blood, urine, cerebral spinal fluid (155, 156, 159), and the pineal gland (156, 160). Synthesis of endogenous DMT begins with the decarboxylation of tryptophan to tryptamine. *N*-methyltryptamine (NMT) and DMT are the products of methyl group additions to tryptamine by the enzyme indolethylamine-*N*-methyltransferase (160). DMT levels were found to increase under stress in the rodent brain and adrenal gland (161). This can activate trace amine-associated receptors and serotonin receptors (5-HT-Rs), such as the 5-HT_1A_-Rs, 5-HT_2A_-Rs, and the 5-HT_2C_-Rs (159, 162). It was suggested that endogenous DMT has a role in cellular protective mechanisms (155).

Exogenous DMT is metabolized by MAO and peroxidases leading to the metabolites NMT, 6-hydroxy-DMT, 6-OH-DMT-*N*-oxide, DMT-*N*-oxide, and indole-3-acetic acid (160). The pharmacokinetics of DMT shows a rapid onset of action within 5–30 min. This is followed by an intense modification of the mental state lasting for approximately 4 h (163). The routes of DMT administration are *via* smoking or snorting. For the hallucinogenic or psychedelic effects to occur, an oral formulation must contain MAOIs to prolong the half-life of DMT in the body. MAOIs block the enzyme in the stomach after which DMT is able to be absorbed through the stomach lining into the blood stream. An oral dosing of DMT, e.g., *via* ayahuasca, produces both behavioral and neuroendocrinological effects, such as a decrease in locomotor activity, cognitive impairments, sympathomimetic effects, increased prolactin, and cortisol levels (164, 165). DMT also interacts with various ionotropic and metabotropic receptors in the glutamate, DA, and acetylcholine systems. The subjective effects of exogenous DMT are primarily mediated by 5-HT_2A_-Rs. 5-HT_2C_-Rs play little or no role (166, 167). Glutamatergic mGluR2 receptors might have modulatory effects in DMT action (167). DMT does not affect DA receptors but may alter DA levels in the brain with subsequent neurochemical and behavioral effects.

Chronic DMT induces tolerance for some behavioral and subjective effects. However, it failed to elicit tolerance to the disruption of responding maintained on a fixed-ratio schedule of food reinforcement (168, 169). DMT yields similar discriminative stimulus effects as the serotonergic hallucinogens 2,5-dimethoxy-4-methylamphetamine (DOM) and LSD. Furthermore, DMT fully substituted in DOM-trained rats and for LSD in rats and pigeons (170, 171).

Beside its sought-after acute effects, DMT can cause considerable side effects. The ingestion of DMT may induce intense fear, paranoia, anxiety, grief, and depression, which may result in physical harm to the user or others (157). There have been no serious adverse events reported on long-term use of DMT apart from the acute cardiovascular effects. Single and repeated administrations of DMT produce marked changes in the cardiovascular system (172). In fact, DMT has been reported to act as neuroprotective agent, working *via* Sigma-1 receptor (Sig-1R) activation (173–177). Sig-1Rs activate the antioxidant response elements (176). Hence, DMT may function as an indirect antioxidant. Frecska et al. (177) have suggested that peripheral synthesis of DMT, consumptions of DMT-containing plant material, or systemic administration of DMT can trigger endogenous central nervous system pathways that produce psychedelic experiences. At the same time, it may serve mechanisms such as neuroprotection and neuroregeneration. Interestingly, ayahuasca and DMT mixtures have been proposed as a treatment for psychiatric disorders. Symptoms of schizophrenia, such as delusions and hallucinations, have been assumed to involve activation of 5-HT_2A_-Rs along with changes in the DA system (166, 178). Endogenous DMT has been reported to be increased in schizophrenic patients during psychotic episodes (179) indicating that the endogenous DMT signaling pathway might be a treatment target for schizophrenia. Based on animal models and on clinical studies in humans, DMT has potential antidepressant and anxiolytic effects (180), possibly mediated by a 5-HT_1A_-R agonistic action (181). Further therapeutic applications include the treatment of cancer and inflammations. DMT has been shown to increase immune system activity (165, 182). Sig-1R activation can reduce pro-inflammatory cytokines and enhance the production of the anti-inflammatory cytokine IL-10 (183).

In conclusion, DMT is a naturally occurring psychoactive compound found in various plants. It is now understood that its main psychoactive effects are mediated by 5-HT_2A_-R activation. Endogenous DMT may play a role in the immunoregulation in peripheral and brain tissues. Preliminary evidence now suggests a possible therapeutic use of DMT.

## Novel Serotonergic Hallucinogens

Since thousands of years, indigene cultures in North and South America have used plants and mushrooms containing serotonergic hallucinogens for shamanic rituals and religious ceremonies (184). The most famous examples are (1) *Psilocybe* mushrooms containing psilocybin, which were used as Teonanacatl (“god’s flesh”) by the Aztecs, (2) the cactus *Lophophora williamsii* enclosing mescaline and applied as Peyote or Peyotl by Mexican and North American indigene cultures, and (3) a brew of *Banisteriopsis caapi* and *Psychotria viridis* called Ayahuasca utilized by Amazonian indigene cultures containing the psychedelic ingredient DMT together with harmala alkaloids acting as MAOIs inhibitors and preventing the metabolism of DMT (185).

Classical serotonergic hallucinogens usually have either a tryptamine or phenylethylamine basic structure (186). Typical tryptamines, such as psilocybin and its psychoactive metabolite psilocin, 5-methoxy-*N*,*N*-dimethyltryptamine (5-MeO-DMT), and bufotenine, resemble in their structure the neurotransmitter 5-HT, while the phenylethylamine mescaline has a similar basic structure as the neurotransmitter DA and as amphetamines. In addition, ergoline alkaloids such as the naturally occurring d-lysergic acid amide, also called ergine—the psychoactive compound of *Turbina corymbosa, Argyreia nervosa*, and *Ipomea tricolor*—and the semi-synthetic LSD (Delysid^®^), have a tryptamine backbone as well (186).

It was suggested that the term “hallucinogens” may be a misnomer as these drugs not necessarily produce real hallucinations, at least when applied at typical doses, but many other emotional, perceptual, cognitive, and behavioral effects. It was suggested that “psychotomimetics” might be the more appropriate term for them (186). However, all 5-HT hallucinogens have in common that they induce altered states of consciousness (186, 187). According to Hollister (188), the psychoactive effects of classical serotonergic hallucinogens usually include (1) somatic symptoms: dizziness, weakness, tremors, nausea, drowsiness, paresthesia, and blurred vision; (2) perceptual symptoms: altered shapes and colors, difficulty in focusing on objects, sharpened sense of hearing, and rarely synesthesia; and (3) psychic symptoms: alterations in mood (happy, sad, or irritable at varying times), tension, distorted time sense, difficulty in expressing thoughts, depersonalization, dream-like feelings, and visual hallucinations.

All tryptamine- and phenylethylamine-based hallucinogens share the agonistic mechanism of action at postsynaptic 5-HT_2A_-Rs and 5-HT_2C_-Rs, where they act as partial, mixed-partial, or full agonists (186, 189). In animals and humans, 5-HT_2A_-R antagonists such as ketanserin are able to block most of the behavioral and psychotropic effects of psilocybin, mescaline, DOI, and LSD, indicating that the 5-HT_2A_-R agonism is necessary for the induction of psychedelic effects (189–194). However, some of these drugs show a strong affinity to 5-HT_1A_-Rs and other 5-HT receptor subtypes as well as to DA D2-Rs. These additional mechanisms are likely to contribute to the specific psychotropic effects of each compound (189, 191, 195). A decade ago, it has been proposed that only 5-HT_2A_-Rs coupled to metabotropic mGluR2 mediate the psychotogenic effects of 5-HT hallucinogens (196)—a position that has been questioned recently (197). At the neuronal level, 5-HT hallucinogens, such as psilocin, LSD, and DMT, directly activate 5-HT_2A_-Rs located on cortical pyramidal neurons. In addition, they increase extracellular glutamate levels in the prefrontal cortex through stimulation of postsynaptic 5-HT_2A_-Rs located on large glutamatergic pyramidal cells in deep cortical layers V and VI. This glutamate release leads to an activation of α-amino-3-hydroxy-5-methyl-4-isoxazolepropionic acid receptors and *N*-methyl-d-aspartic acid (NMDA) receptors on cortical pyramidal neurons (187).

Historically, LSD was probably one of the first NPS of the hallucinogen class as it was a semi-synthetic compound whose psychedelic effects have only accidentally been discovered by its inventor Albert Hofmann in 1943 (198). The next, even though less accidental, producer of NPS hallucinogens was Alexander T. Shulgin, who synthesized hundreds of novel hallucinogenic tryptamines and phenylethylamines in his home laboratory. He described the synthesis of these compounds and also their psychotomimetic effects experienced in self-experiments in detail in his books PIHKAL and TIHKAL (199, 200). He created several dimethoxy-substituted phenylethylamines, such as DOM, 2,5-dimethoxy-4-bromoamphetamine (DOB), 2,5-dimethoxy-4-iodoamphetamine (DOI), and 2,5-dimethoxy-4-ethylamphetamine (DOET), which all display strong hallucinogenic properties. These drugs usually have much longer durations of action (12–30 h) and are much more potent agonists at 5-HT_2A_-Rs (50- to 175-fold) compared to their related phenylethylamine derivative mescaline (duration of action: 4–8 h) (189, 199, 200). Also, another novel class of substituted dimethoxyphenethylamines—the “2C psychedelics”—was invented by Shulgin, which mostly contains methoxy groups at positions 2 and 5 of a benzene ring together with lipophilic substituents (often halogens) at position 4. The most famous exponent of this class is 2,5-dimethoxy-4-bromophenethylamine (2C-B, “nexus,” “bromo”), which was initially marketed as a legal surrogate of MDMA (“ecstasy”) in the late 80s before it was finally scheduled by the UN Commission on Narcotic Drugs in March 2001 (201). Dozens of 2C-B analogs, such as 2C-I, 2C-C, 2C-F, 2C-E, and 2C-N, have later been sold as “research chemical” or “legal highs” *via* the Internet. Because their structure can be easily changed without losing their psychoactive properties, 2C drugs have, thus, often been referred as a typical class of designer drugs (201). 2C drugs commonly do not only act as 5-HT_2A_-R and 5-HT_1A_-R agonists but also as monoamine transporter inhibitors (195). Consequently, these compounds have not only hallucinogenic properties but also slight stimulating and empathogenic/entactogenic effects sometimes mimicking the effects of the prototypical empathogen MDMA (199). Shulgin also described novel ergolines such as *N*-allyl-nor-lysergic acid diethylamide (AL-LAD), *N*-ethyl-nor-lysergic acid diethylamide (ETH-LAD), and *N*-propyl-nor-lysergic acid diethylamide (PRO-LAD) (200). These LSD-analogs are as potent as LSD (potency relative to LSD in human: AL-LAD: 110%, ETH-LAD: 140%, PRO-LAD: 90%), but AL-LAD and PRO-LAD have shorter duration of action (6–8 h) as ETH-LAD and LSD (both: 8–12 h) (189, 200). Finally, Shulgin synthesized a large number of novel tryptamines, such as 4-hydroxy-*N*-methyl-*N*-ethyltryptamine (4-HO-MET), 5-methoxy-diisopropyltryptamine (5-MeO-DIPT), and alpha-ethyltryptamine (alpha-ET), which are mostly hallucinogenic, but with some exceptions (e.g., alpha-ET has pronounced empathogenic effects) (200). Shulgins books PIHKAL and TIHKAL served as cook books for a generation of illegal drug laboratories. His dimethoxyphenethylamines, 2C drugs, and novel ergolines and tryptamines are still circulating as NPS, although they have been created at least 20 years ago. However, their human toxicology and their consequences are still unknown as they are neither used frequently nor purely enough in order to systematically investigate their chronic effects in recreational users.

In the last decade, a substantial amount of new serotonergic hallucinogens appeared on the drug markets. As their number grows each day, it is simply not possible to list them exhaustively here. Thus, only some prototypical exponents of each class will be discussed. Again the main classes are either tryptamines and related ergolines or substituted phenethylamines but also some new classes such as benzodifurans and aminoindanes occurred (202–205). Novel tryptamines such as alpha-methyltryptamine (AMT), *N*,*N*-diallyl-5-methoxytryptamine (5-MeO-DALT) have multiple serotonergic actions including strong affinity for the 5-HT_2A_-R, but can also act as monoamine transporter substrates. They combine hallucinogenic effects with stimulant and empathogenic features (203, 205). Novel ergolines such as 1-propionyl-lysergic acid diethylamide (1P-LSD) and lysergic acid 2,4-dimethylazetidide (LSZ) are LSD-analogs mainly interacting with 5-HT_2A_-R and 5-HT_1A_-R subtypes. They are slightly more potent as LSD and have a comparable duration of action. They are also mostly marketed as blotters (202, 205). *N*-2-methoxybenzyl derivatives of 2,5-dimethoxy-substituted phenethylamines also called NBOMe drugs, such as 25B-NBOMe, 25C-NBOMe, 25I-NBOMe, 25T2-NBOMe, and mescaline-NBOMe, are highly potent 5-HT_2A_-R full agonists. In addition, they show a high-binding affinity to the 5-HT_1A_-R, to adrenergic α1A and α2A, and histamine H1 receptors. Some derivatives also possess low-to-moderate affinity to DA D2- and D3-Rs. Several NBOMe drugs show higher affinity, higher activation potency, and higher activation efficacy at 5-HT_2A_-Rs than LSD. Anecdotal user reports consider them as very strong hallucinogens (195, 205, 206). Benzodifurans, the so-called “fly drugs,” such as 2C-B-FLY, 3C-Bromo-Dragonfly, and TFMFly, are a group of ring-substituted phenethylamines that are structurally related to MDMA. Unlike MDMA, benzodifurans commonly display a high affinity for 5-HT_1A_-Rs, 5-HT_2A_-Rs, 5-HT_2B_-Rs, and 5-HT_2C_-Rs, but show only little action at monoamine transporters (195, 205). Aminoindanes, such as 5-iodo-2-aminoindane (5-IAI), are usually 5-HT and noradrenaline (NA) releasers that have been sold as a legal surrogate for MDMA (203, 205). At least 5-IAI was recently demonstrated to show a strong affinity for 5-HT_1A_-Rs and 5-HT_2A_-Rs, thus, indicating that aminoindanes can not only be empathogens, but they can also display hallucinogenic properties (207).

At the moment, systematic investigations on the prevalence of novel serotonergic hallucinogens are rare. In the global drug survey of 2012, 11.3% of the respondents, mainly regular drug users, reported to have used a 2C drug at least once during their lifetime and that 2C-B was the most common one (8.4%). Moreover, 2.6% of respondents reported to have used 25B-NBOMe, 25C-NBOMe, or 25I-NBOMe at least once, while 25I-NBOMe (2.0%) was the most popular derivate. The most common drug source for NBOMes was the Internet (41.7%). For comparison, 39.4% of the respondents in this survey had used LSD and 43.1% “magic mushrooms” at least once during lifetime (206). A recent representative survey in the US (*N* = 213.076) revealed that the lifetime prevalence of novel hallucinogenic drugs was generally low: NBOMes, 0.015%; 2C drugs, 0.195%; dimethoxyphenethylamines, 0.019%; novel tryptamines, 1.060% (primarily DMT) (208). It should be noted that DMT was the only hallucinogenic NPS that was systematically asked for but that participants were given the opportunity to type in the names of NPS they used, indicating that these numbers are likely underestimated (208).

Data from the European Drug Emergencies Network have recently shown that, compared to all other investigated drugs, novel tryptamine users have the highest risk [odds ratio (OR) = 12.4] to be treated for psychosis-like symptoms in an emergency care unit, while also LSD use was significantly associated with an increased psychosis risk (OR = 3.1) (209). Overall frequencies for the development of acute psychosis following experimentally administered LSD range between 0.08 and 4.6%, while patients having a psychiatric disorder before LSD intake displayed the highest risk (185). However, if 5-HT hallucinogens can also induce long-lasting psychotic disorders is still controversially discussed (185). Beyond acute psychotic reactions including hallucinations, ego impairment, and paranoia, also “bad trips,” panic attacks, confusion, agitation, aggression, and disorientation are common acute psychiatric side effects of classical and novel serotonergic hallucinogens (185, 203, 205, 210). Moreover, also nausea and vomiting, serotonin syndrome including hyperthermia, liver and kidney failures, and cardiovascular complications have been reported for serotonergic hallucinogens. The acute toxicity of high potency dimethoxyphenylethylamines, NBOMEs, and 2C drugs seems to be considerably increased compared to classical hallucinogens. High potency compounds have been associated with a number of life-threatening conditions, such as rhabdomyolysis, seizures, vasoconstriction/hypertension, tachycardia, pulmonary edema, and serotonin syndrome with hyperthermia and organ failures, sometimes with fatal outcome (210–214).

Chronic side effects of hallucinogens can include panic disorder and a hallucinogen persisting perception disorder (HPPD, “flashback”) (185). In fact, 60% of LSD users know “flashbacks” and 4% of users report sustained HPPD of putative clinical significance (215). Also, MDMA users are at risk to develop HPPD (216). It is highly likely that potent novel serotonergic hallucinogens bear a strong risk to induce HPPD too. Changes of 5-HT_2A_-R function in the visual cortex were claimed to be responsible for HPPD (185, 216). In general, 5-HT-Rs show considerable plasticity after exposure to serotonergic drugs. Accordingly, due to post-transcriptional mechanisms, 5-HT_2A_-Rs show a rapid and long-lasting downregulation in response to 5-HT agonists (217–219). Specifically, LSD, 2-bromo-LSD, and DOI selectively reduce 5-HT_2A_-R density without affecting 5-HT_2C_-Rs (220). Furthermore, hallucinogens acting at 5-HT_2A_-Rs show strong behavioral tolerance coinciding with a robust decrease in 5-HT_2A_-Rs. This might explain the strong tolerance effect of 5-HT hallucinogens (221). Recently, it was shown that 5-HT hallucinogens can also reduce either 5-HT_2A_-R binding sites or glutamate-binding sites and that tolerance effects were correlated with changes in both binding sites (222).

High potency 5-HT hallucinogens—specifically if they have a long duration of action—are probably neurotoxic due to their sustained activation of 5-HT_2A_-Rs that can induce apoptosis in neurons (223). Neurotoxic effects have been shown not only for DOI (224, 225) and 5-MeO-DIPT (226) but also for chronic low doses of LSD (227) and repeated high doses of MDMA (223–225). Thus, it is likely that all long-acting dimethoxyphenylethylamines, 2C drugs, NBOMes, tryptamines, and ergolines with strong agonistic actions at 5-HT_2A_-Rs have a neurotoxic potential.

In conclusion, beyond LSD, mescaline, and psilocybin, a vast amount of new serotonergic hallucinogens appeared on the drug market during the last decades. Their distribution has strongly increased and will likely further increase in the future due to their easy availability on the Internet. Alarmingly, little is known about the acute and chronic effects of novel 5-HT hallucinogenic drugs in human users. The neuropsychiatric long-term consequences of regular intake of such compounds are completely unclear. However, it is becoming increasingly apparent that high potency drugs with very strong affinities to 5-HT_2A_-Rs and long durations of action bear a considerable risk for negative health effects and fatalities.

## Mephedrone and Methylone

Dozens of research chemicals with a cathinone basic structure appeared as “legal highs” on the drug marked. However, an exhaustive discussion of all of them is not possible here due to space restrictions (228). Thus, in this section, the two generic compounds, mephedrone and methylone, are discussed as important examples.

Mephedrone (4-methylmethcathinone) is a substituted cathinone homolog of ephedrine first described in 1929 (229, 230). Mephedrone has a ring-substituted cathinone structure which is related to the phenethylamine family, to which also drugs such as amphetamine, MDMA, and methamphetamine belong to (231). As a hydrochloride salt, mephedrone is a water soluble white, yellow, beige, or brown powder. In the European market, it is sold under different names such as Meow Meow, Bubbles, Mef, MMC Hammer, and many more (231). Mephedrone is available on the Internet, or from street dealers. On Internet sources, mephedrone is often marketed as bath salt, plant fertilizer, or research chemical (232, 233).

Mephedrone was first identified as an abused drug by European authorities in 2007 (234, 235). By 2010, mephedrone use spread, and the drug was found in many European countries (236). The use of mephedrone increased rapidly in the club scene and soon reached the level MDMA and cocaine use, reaching a life-time use in Europe among the 15- to 24-year olds of 6% by 2010 (236, 237). Mephedrone is frequently used together with other synthetic cathinones, such as methylone, butylone, or ethylcathinone (236). The predominant user populations are teenagers and young adults (238), thereby use of new psychoactive cathinones is highly correlated with binge-drinking habits in young adults (239).

Mephedrone can be consumed by different routes. In an oral preparation, mephedrone powder is rolled up in cigarette paper (bombing). Furthermore, intranasal, intramuscular, intravenous (slamming), and rectal routes of administration have been reported (240). Mephedrone is also mixed with other drugs, such as heroin, alcohol, cocaine, MDMA, or cannabis (235, 241). Consumption usually takes place in a social context at home, at rave parties, clubs, or music festivals. Mephedrone binge consumption has been reported to last for up to 9 h with a new dose all 0.5–2 h (231). Intranasal mephedrone elicits rapid effects within minutes. They reach a peak level in less than 30 min and last for up to 1 h. Orally applied mephedrone powder or tablets induce psychoactive effects in 45–120 min which may last for 2–4 h, thereby a sequence of first intranasal snorting followed by repeated oral ingestion has been reported, in order to achieve both, fast and long-lasting effects (231, 240, 242, 243). The sought-after psychoactive effects of mephedrone comprise an elevated mood, the feeling of an intense euphoria, a sense of well-being, increased self-esteem, motor excitation, reduced tiredness, increased alertness and concentration, talkativeness, empathy, disinhibition, and a mild sexual stimulation (231, 244, 245).

A high dose and/or chronic consumption of mephedrone have been associated with significant adverse effects. Those include cardiovascular, gastrointestinal, and neurological side effects (233, 246). Well-described effects are also jaw clenching, reduced appetite, increased body temperature, increased sweating, abnormal vision, dilated pupils, headaches, tachycardia, palpitations, hypertension, arrhythmias, chest pain, nausea, bruxism, teeth grinding (bruxism), rhabdomyolysis, and renal failure (247). An important dangerous side effect is the significant hyponatremia. This is similar to that shown after acute MDMA consumption. It is supposed to be induced by a combination of sweating, electrolyte loss, and antidiuretic hormone secretion (247). The intranasal application of mephedrone is associated with a significant nasal irritation. Mephedrone addiction is often associated with intravenous drug use that is also found to be linked to an increased risk of using other addictive drugs (248). Intravenous mephedrone injections often result in vein blockages, leading to localized infections, blisters, abscesses, scabs, lumps, gangrenous tissue, blood clots, and large necroses at the injection site (249). Major adverse psychiatric effects associated with mephedrone use include agitation, anxiety, dysphoria, depression, insomnia, hallucinations, paranoia, delusions, aggressive behavior, as well as suicidal ideation and suicidal action. Cognitive impairments affect short-term memory and attention span (250). Psychotic effects predominantly occur after a high mephedrone dose, after binge consumption in one session, and in users with an individual vulnerability for psychiatric disorders (251–253). Fatalities resulting from mephedrone use have been reported worldwide now (254). They are related to hyponatremia and brain edema (255–257). However, the lethal dose (LD_50_) is not known yet (258).

Accumulating evidence suggests that mephedrone has a clear addiction potential (246, 259, 260). The abuse potential for intranasally consumed mephedrone was suggested to be comparable with that of cocaine or methamphetamine (246). Among regular users, about 50% reported an addiction to the drug (261) and about 25% admitted mephedrone-related craving (262). Mephedrone withdrawal effects include tiredness, insomnia, impaired concentration, irritability, tremor, temperature dysregulation, palpitations, headaches, depression, anxiety, and paranoia (235, 244, 260).

Virtually all synthetic cathinones are considered to inhibit the monoamine uptake in the brain, thereby mephedrone acts as a substrate for the transporter proteins and evokes a reverse neurotransmitter transport and, thus, neurotransmitters release (231, 244, 263).

Synthetic cathinones including mephedrone are now classified as illicit substances in many countries (231). However, since the legal ban of single substances came in place, various second-generation analogs have appeared, including 4-methyl-*N*-ethylcathinone (4-MEC). The consumption may in the long term only effectively be limited when whole substance classes, i.e., with a cathinone lead structure, are legally controlled (231).

Methylone (3,4-methylenedioxymethcathinone) is a substituted cathinone methylated on the amine group of the keto-phenethylamine backbone. It has a chemical structure similar to that of MDMA by a methylenedioxy ring attached to the aromatic ring (264). Methylone was first synthesized in 1996 as a potential antidepressant and anti-Parkinson agent (265), which, however, never made it into pharmacotherapy. Instead, it emerged on the street market under different names, such as Ease, Explosion, M1, MDMC, and bk-MDMA (231, 246). Methylone was marketed initially in a liquid solution as a vanilla-scented room odorizer. Following its introduction in 2004, methylone could be purchased in the Internet and in headshops (266), where it was sold in powder form and as tablets (267). Methylone use has been reported to be high in the club scene (261) and in addicts on substitution therapy (267).

Similar to other cathinones, methylone can be administered by different routes, such as orally, intranasally, intravenously, sublingually, or rectally. The most popular route is the oral administration. A common application pattern is to start with a large “boosting” dose and then maintain effects by smaller “bumping” doses (268, 269). The onset of the desired psychoactive effects of methylone is usually 15–60 min after oral administration. These effects last approximately 30–45 min (268). They have been described as an amphetamine-like stimulation with calm euphoria, happiness, thought acceleration, alertness, restlessness, reduced fatigue, and increased locomotor activity. They might also involve MDMA-like entactogenic effects with a strong sense of emotional openness, enhanced empathy, and reduced fear (270). A methylone high can be from moderate to extreme euphoria with tingling sensation (231, 268).

The adverse effects of methylone include anxiety and psychosis with derealization, depersonalization, hallucinations, and suicidal ideation. Cognitive impairments affect the short-term memory (258). Furthermore, methylone may induce seizures and hyponatremia, similar to that induced by MDMA. Methylone may also induce a hyperthermia (271). This is believed to be a major cause for the fatal consequences of a methylone overdose (272). Other factors in fatal overdose can be cardiac events, metabolic acidosis, rhabdomyolysis, acute renal failure, intravascular coagulation, and a serotonin syndrome (273–276).

Accumulating evidence suggests a considerable addictive potential of methylone (231, 277). Much like mephedrone, methylone acts as a monoamine reuptake blocker that leads to a profound hyperactivity of DA, 5-HT, and NA in the brain and periphery (263). In particular, dopaminergic and serotonergic adaptations in the brain may drive the addiction potential of psychostimulant drugs (278, 279). The use and abuse of the substance emerged with considerable side effects around the world (268). The legal ban of methylone started in 2007 with now an increasing number of countries controlling it (231).

## Ketamine and Novel Dissociative Drugs

(±)Ketamine (±2-chlorophenyl-2-methylamino-cyclohexanone) is a non-competitive antagonist of the NMDA receptor (27). It has been widely used in clinical settings as an anesthetic agent and in veterinary medicine. However, ketamine is also recreationally consumed in entertainment settings for its hallucinogenic, mood enhancing, and reinforcing properties by young club goers (28, 280–282). Ketamine is a derivative of phencyclidine (PCP), which was discovered as anesthetic in 1956 and became a popular street drug during 1960s (280). Ketamine is regulated in many countries due to its abuse potential as a psychotropic substance (282). A significant number of studies have demonstrated that ketamine has a short-acting antidepressant effect and is increasingly used to treat therapy-refractory major depression and pain (29, 283–285). Although ketamine is viewed as a safe substance in medical settings, its recreational use is reported to impose adverse effects on users by producing neurological and peripheral toxicity (286, 287).

Ketamine can be administered through intravenous, intramuscular, smoking, and snorting routes (288). Apparently, snorting or intranasal use is the main route of ketamine consumption among recreational users (289). Ketamine produces dose-dependent effects. Lower doses are associated with a feeling of relaxation. At higher doses, ketamine induces a dream-like state called a “*k-hole*.” This experience is akin to dissociative anesthetic characteristics (290, 291). Chronic ketamine use is reported to induce schizophrenia-like positive and negative symptoms, including hallucinations, detachment, delusion, auditory, and verbal hallucinations (292). A major concern of ketamine use is that people drive under the influence of the drug (293). Ketamine can impair cognitive functioning, such as executive and memory function, as well as attentional control (294, 295). Ketamine users are also more vulnerable to HIV infections. The use of the drug is reported to enhance sexual experience and predispose users to engage in unprotected sex (296, 297). Ketamine-related mortality has increased 10-fold in the UK from 1999 to 2008 (287), while in Australia 40% of party drug users were tested positive for ketamine use (281).

Some of the most common complaints of ketamine use include chest pain, palpitations, and tachycardia (298). However, these symptoms are often transient (286). Abdominal pain and urinary tract symptoms, such as suprapubic pain, dysuria, and hematuria, are common symptoms of chronic regular ketamine use (299–301). Findings from clinical case studies have shown that ketamine use can decrease bladder volume, bladder wall thickening, mucosal enhancement, dilation of ureter, and cause perivesical inflammation (302, 303). The renal toxicity of ketamine is due to the direct toxic action of ketamine and its metabolites (288).

Fatigue, poor appetite, drowsiness, craving, anxiety, sleeping problems, and dysphoria are common physical and psychological side effects of ketamine use (304, 305). Currently, there is no specific treatment for ketamine users presenting with peripheral toxicity. However, it was reported that cessation from ketamine abuse may lead to a recovery from organ damage (28). Despite its abuse potential and reported side effects, ketamine has promising medicinal properties. Currently, it is used to treat therapy-refractory depression (306), although the antidepressant effect of a single infusion only last for some days. Despite that development, which moved the drug increasingly out of the drug abuse focus, proper prevention strategies for young club goers engaged in recreational ketamine use are still warranted. Moreover, addiction experts warned recently that psychiatrist should not underestimate the addictive potential of ketamine when treating depressive patients with the drug (307).

Dissociative anesthetics such as PCP and ketamine are non-medically used since more than 60 years (280). Importantly, “dissociative anesthetics” are originally defined as substances inducing a general form of anesthesia characterized by analgesia, amnesia, and cataplexy, but with minimal effect on respiratory function (308). Today, the term “dissociative drugs” includes the family of dissociative anesthetics but is not restricted to them. It more generally denotes hallucinogenic drugs inducing dissociative states, including sensory alterations and hallucinations as well as dream-like states or trance (280). More than 14 known derivatives of PCP have been marketed for non-medical but also illicit use already between the late 1960s and the 1990s. However, with the advent of online drug shops selling “legal highs,” novel dissociative drugs appeared too. Starting with the first dissociative, 4-MeO-PCP in 2008, thenceforth at least 12 novel dissociative drugs appeared on the drug marked, which were unknown in the scientific literature prior to their introduction to the drug market (280). In the meantime, the most common agents, methoxetamine (MXE), diphenidine, methoxphenidine (MXP), 3-MeO-PCP, and 4-MeO-PCP, have reached widespread use in Europe and North America.

PCP, ketamine, and its novel derivatives belong to the chemical class of arylcyclohexylamines, which have in common that they act as non-competitive antagonists at the PCP-binding site of the NMDA receptor (280). Beyond their high affinity for NMDA receptors, some of the arylcyclohexylamines have shown agonistic actions at DA receptors (e.g., D2 receptors) and inhibitory effects at DA transporters, agonistic effects at μ-opioid and σ-1 receptors, as well as antagonistic actions at both nicotinic and muscarinic acetylcholine receptors. It is plausible that the specific receptor profile of each compound mediates its characteristic psychotropic effects (280). Beyond the desired dissociative acute effects, these drugs exert a number of severe and sometimes fatal side effects. Following MXE ingestion, users were confused, agitated, hallucinating, and unresponsive. The somatic and neurological adverse effects included tachycardia, hypertension, ataxia, mydriasis, nystagmus, seizures, leukocytosis, massive rhabdomyolysis, hepatic failure, onset of acute renal failure, sinus bradycardia, elevated creatinine kinase, and hyponatremia (210). Several fatalities have been reported each for MXE, MXP, 3-MeO-PCP, and 4-MeO-PCP (210, 240). According to anecdotal reports, MXE and MXP seem to have stronger empathogenic and euphorigenic properties than PCP and ketamine (210).

Novel dissociative drugs from the arylcyclohexylamine class, such as MXE, have been sold as a “legal” and “bladder friendly” alternative to ketamine. However, animal studies have shown that MXE and likely all arylcyclohexylamines are in fact equally toxic for the bladder and the kidneys as ketamine when applied chronically (240). Further chronic side effects of novel arylcyclohexylamines have not been investigated yet, but it is likely that the total class might have an addictive potential similar to that of ketamine and PCP (309, 310). This seems to be specifically high in adolescents and young adults (311). Moreover, like PCP and ketamine, all arylcyclohexylamines with a strong action at the NMDA receptor may impair memory function (310) and induce psychotic symptoms after acute and chronic consumption (312, 313).

## γ-Hydroxybutyrate, γ-Butyrolactone, and 1,4-Butanediol

γ-Hydroxybutyrate (GHB, or sodium oxybate), γ-butyrolactone (GBL), and 1,4-butanediol (1,4-BD) are potent central depressant agents with a broad spectrum of subjective, behavioral, and neuropharmacological effects in humans. These drugs are used clinically for the treatment of neuropsychiatric disorders such as narcolepsy, alcohol withdrawal, and fibromyalgia but also instrumentalized illicitly for hedonic purposes (314).

GBL and 1,4-BD are rapidly metabolized endogenously to GHB. The psychoactive effects of the drug result from this conversion (315, 316). GHB is an endogenous short-chain fatty acid. It is biosynthetically derived from γ-aminobutyric acid (GABA) which occurs naturally in the mammalian brain, mainly in the hypothalamus and the basal ganglia (317, 318). The molecule binds to GABA-B receptors (319) and to specific GHB receptors (320). Due to the presence of endogenous GHB in the brain, specific G-protein-coupled GHB receptors, and the specificity of the GHB antagonist NCS-382, GHB is considered to be a neurotransmitter (321). While physiological concentrations of GHB seem to be insufficient to stimulate GABA-B receptors, the subjective and behavioral effects of the exogenously applied drug, and thus GBL and 1,4-BD, result from direct stimulation of these receptors (322). Moreover, GHB has extensive downstream effects on DA, 5-HT, NA, glutamate, and acetylcholine transmission (323). GHB, GBL, and 1,4-BD are well absorbed orally in humans. Peak plasma concentrations are reached within 25–60 min, with a half-life of 20–60 min (324, 325). All compounds are metabolized to water and carbon dioxide through the citric acid cycle (326).

In humans, the spectrum of the subjective effects of these compounds ranges from euphoria, stimulation, and disinhibition in oral doses of 10–25 mg/kg (327–329), toward heavy sedation and loss of consciousness at oral doses of 35–70 mg/kg (324, 330). A seemingly paradoxical pattern of concomitant sedation and stimulation was described in several reports (327, 328).

GHB, GBL, and 1,4-BD strongly influence behaviors related to core autonomic functions, such as the control of food intake, sexual behavior, and sleep–wake regulation (314). GHB was reported to normalize dysfunctional food intake behavior and body weight in preclinical and in clinical studies (331–334). It was effective in the treatment of binge-eating disorder (335). Confirming subjective reports from illicit GHB users (336–338), the drug was experimentally shown to have prosocial (328), and prosexual effects in healthy male subjects (339). Moreover, GHB and its precursors have a unique effect on sleep–wake regulation (340). Since GHB improves sleep and daytime vigilance, it is used as standard treatment for disorders of the sleep–wake cycle, such as narcolepsy and fibromyalgia (341–343).

Neuropharmacological studies with GHB, GBL, and 1,4-BD are scarce and were until recently limited to early EEG investigations. Resting state EEG studies showed a paradoxical EEG-behavioral dissociation with the occurrence of increased delta and theta oscillations, during wake states, which usually occur during sleep (344, 345). Moreover, increased nocturnal slow wave sleep under the influence of GHB was demonstrated (346). A recent EEG study showed increased current source density of theta oscillations in the posterior cingulate cortex and alpha oscillations in the anterior cingulate cortex (ACC) under 20 and 35 mg/kg GHB in healthy male subjects (347). In the first functional neuroimaging study with GHB, 35 mg/kg of the drug increased regional cerebral blood perfusion in the ACC and the insula, both of which correlated with increased subjective ratings of emotion and body awareness (348). Moreover, the drug increased the susceptibility of the mesolimbic reward system, resulting in an increased sexual arousal after the presentation of erotic but also neutral pictures of persons. This effect correlated with an increased activity in the nucleus accumbens and the ACC (349).

The euphoric, prosocial, and prosexual effects of GHB, GBL, and 1,4-BD are instrumentalized illicitly, mostly by members of urban subcultures (314). Internationally, GHB, GBL, and 1,4-BD are mainly used as recreational drugs by young adults aged 20–29 years (349, 350). Reliable prevalence data are difficult to obtain (351). However, the prevalence of GHB, GBL, and 1,4-BD seem low compared to other drugs of abuse and are estimated at about 4.3% in Europe (349). After GHB was used in a deadly case of drug-facilitated sexual assault in the USA in the year 2000, the drug was internationally banned (352). However, a recent meta-analysis showed that GHB is very infrequently used as a date rape drug (353).

The development of addiction after illicit use of these drugs was estimated for about 4–21% of illicit users (351). Both addiction and withdrawal can be severe and in extreme cases lead to psychosis, delirium, and death (314). Interestingly, the development of addiction after medical use of GHB is at a very low rate with an estimated risk of about 0.015% (351).

Internationally, GHB is approved for the treatment of narcolepsy with cataplexy. In a recent meta-analysis, it was confirmed to be effective in treating major, clinically relevant narcolepsy symptoms and sleep architecture impairments in patients (354). Another clinical indication is the treatment of alcohol withdrawal, for which GHB is used since two decades in Italy and Austria (355). Moreover, several randomized controlled trials showed a therapeutic effect of GHB on clinical course and life quality in patients suffering from fibromyalgia (343, 356–358). Other neuropsychiatric disorders in which GHB showed therapeutic effects are binge-eating disorders (335), schizophrenia (359), Parkinson’s disease (360), and cluster headache (361, 362), mostly by regulating homeostatic dysbalances, as well as improving sleep and pain symptoms. Because disrupted homeostatic processes including food intake, sexual behavior, and the sleep–wake cycle frequently occur in major depressive disorder, GHB was proposed as an experimental therapeutic in this condition (363, 364). However, therapeutic use of the drug is limited by side effects, such as nausea, vomiting, altered consciousness, and nocturnal O_2_ desaturations (357, 365–367).

In conclusion, GHB and partially its precursors GBL and 1,4-BD have undeniable caveats such as limiting side effects and abuse liability. These, however, seem to be outweighed by a unique spectrum of clinically relevant psychopharmacological effects, which warrant further studies in neuropsychiatric conditions such as major depressive disorder following a personalized treatment paradigm (368).

## Conclusion

The amount of evidence on the psychoactive drugs discussed shows that many of them are not really novel anymore. For most of them a classification in terms of their use and harm potential has been made. In fact, most of them are already legally controlled or banned in certain countries. An important feature of this process is that it appears socio-geographically biased. Even in a globalized world, new psychoactive drugs emerge and spread in a regionally bound way. This brings about that evidence on their use, instrumentalization, and abuse accumulates often only regionally. Also, the drug may for a long time not spread beyond the socio-geographic boundaries. However, this does not mean that it is not eventually “discovered” by other societies making use of the drug for a new and essentially different purpose. The scientific challenge is then to use locally gathered knowledge to be prepared for a drug that is novel in a certain culture and establish a judgment on its harm potential and/or medical use on a rather global scale. It also means to delineate future research needs for those drugs that are brand new as a psychoactive drug. This review shows that despite accumulating evidence, for many of those NPSs, a final classification is still in progress and gathering of more evidence is pivotal.

## Author Contributions

CM and ZH planned the review article and contributed to several chapters. CM prepared the final version of the manuscript. CM, ZH, OB, DS, SN, BK, ES, JK, and BQ wrote single chapters of the manuscript.

## Conflict of Interest Statement

The authors declare that the research was conducted in the absence of any commercial or financial relationships that could be construed as a potential conflict of interest.

## References

[1] Jay M High Society. Mind-Altering Drugs in History and Culture. London: Thames & Hudson (2010).

[2] Wadley G. How psychoactive drugs shape human culture: a multi-disciplinary perspective. Brain Res Bull (2016) 126(Pt 1):138–51. 10.1016/j.brainresbull.2016.04.008 27083300

[3] Hagen EH Sullivan RJ Schmidt R Morris G Kempter R Hammerstein P. Ecology and neurobiology of toxin avoidance and the paradox of drug reward. Neuroscience (2009) 160:69–84. 10.1016/j.neuroscience.2009.01.077 19233250

[4] Dellu F Piazza PV Mayo W Le Moal M Simon H Novelty-seeking in rats – biobehavioral characteristics and possible relationship with the sensation-seeking trait in man. Neuropsychobiology (1996) 34:136–45. 10.1159/000119305 8916071

[5] Bidwell LC Knopik VS Audrain-McGovern J Glynn TR Spillane NS Ray LA Novelty seeking as a phenotypic marker of adolescent substance use. Subst Abuse (2015) 9:1–10. 10.4137/SART.S22440 26106262 PMC4472033

[6] Müller CP Schumann G Drugs as an instrument: a new framework for non-addictive psychoactive drug use. Behav Brain Sci (2011) 34(6):293–310. 10.1017/S0140525X11000057 22074962

[7] Müller CP Schumann G. To use or not to use: expanding the view on non-addictive psychoactive drug consumption and its implications. Behav Brain Sci (2011) 34(6):328–47. 10.1017/S0140525X1100135X 22379623

[8] Müller CP Non addictive drug use: the way forward. In: Wolff K White J Karch S, editors. The SAGE Handbook of Drugs & Alcohol Studies – Biological Approaches. (Vol. 2, Chap. 42), London: SAGE (2017). p. 411–34.

[9] Brand R Wolff W Ziegler M. Drugs as instruments: describing and testing a behavioral approach to the study of neuroenhancement. Front Psychol (2016) 7:1226. 10.3389/fpsyg.2016.01226 27582720 PMC4987358

[10] McCreary AC Müller CP Filip M Psychostimulants: basic and clinical pharmacology. Int Rev Neurobiol (2015) 120:41–88. 10.1016/bs.irn.2015.02.008 26070753

[11] Müller CP. Episodic memories and their relevance for psychoactive drug use and addiction. Front Behav Neurosci (2013) 7:34. 10.3389/fnbeh.2013.00034 23734106 PMC3661997

[12] Ahmed SH. Toward an evolutionary basis for resilience to drug addiction. Behav Brain Sci (2011) 34:310–1. 10.1017/S0140525X11000677 22074963

[13] Chick J. Can light or moderate drinking benefit mental health? Eur Addict Res (1999) 5:74–81. 10.1159/000018969 10394037

[14] Heath DB Drinking Occasions: Comparative Perspectives on Alcohol and Culture. Philadelphia: Brunner/Mazel (2000).

[15] Peele S Brodsky A. Exploring psychological benefits associated with moderate alcohol use: a necessary corrective to assessments of drinking outcomes? Drug Alcohol Depend (2000) 60:221–47. 10.1016/S0376-8716(00)00112-5 11053757

[16] Lende DH Leonard T Sterk CE Elifson K Functional methamphetamine use: the insider’s perspective. Addict Res Ther (2007) 15(5):465–77. 10.1080/16066350701284552

[17] Spear LP. The adolescent brain and age-related behavioral manifestations. Neurosci Biobehav Rev (2000) 24:417–63. 10.1016/S0149-7634(00)00014-2 10817843

[18] Kuntsche E Knibbe R Gmel G Engels R. Who drinks and why? A review of socio-demographic, personality, and contextual issues behind the drinking motives in young people. Addict Behav (2006) 31:1844–57. 10.1016/j.addbeh.2005.12.028 16460883

[19] Müller CP Kalinichenko LS Tiesel J Witt M Stöckl T Sprenger E Paradoxical antidepressant effects of alcohol are related to acid sphingomyelinase and its control of sphingolipid homeostasis. Acta Neuropathol (2017) 133(3):463–83. 10.1007/s00401-016-1658-6 28000031 PMC5325869

[20] Bonn-Miller MO Zvolensky MJ Bernstein A Marihuana use motives: concurrent relations to frequency of past 30-day use and anxiety sensitivity among young adult marihuana smokers. Addict Behav (2007) 32:49–62. 10.1016/j.addbeh.2006.03.018 16647822

[21] Fattore L Fratta W. Beyond THC: the new generation of cannabinoid designer drugs. Front Behav Neurosci (2011) 5:60. 10.3389/fnbeh.2011.00060 22007163 PMC3187647

[22] United Nations Office on Drugs and Crime. Single Convention on Narcotic Drugs. (1961). Available from: http://www.unodc.org/unodc/en/treaties/single-convention.html

[23] United Nations Office on Drugs and Crime. Convention on Psychotropic Substances. (1971). Available from: http://www.unodc.org/unodc/en/treaties/psychotropics.html

[24] Schifano F Orsolini L Duccio Papanti G Corkery JM Novel psychoactive substances of interest for psychiatry. World Psychiatry (2015) 14:15–26. 10.1002/wps.20174 25655145 PMC4329884

[25] Singh D Müller CP Vicknasingam BK. Kratom (*Mitragyna speciosa*) dependence, withdrawal symptoms and craving in regular users. Drug Alcohol Depend (2014) 139:132–7. 10.1016/j.drugalcdep.2014.03.017 24698080

[26] Frederiksen NJ Bakke SL Dalum P “No alcohol, no party”: an explorative study of young Danish moderate drinkers. Scand J Public Health (2012) 40:585–90. 10.1177/1403494812458988 23027894

[27] Kornhuber J Weller M Predicting psychotomimetic properties of PCP-like NMDA receptor antagonists. In: Fog R Gerlach J Hemmingsen R Krogsgaard-Larsen P Thaysen Schizophrenia JH, editors. Schizophrenia – An Integrated View. Alfred Benzon Symposium 38. Copenhagen: Munksgaard (1995). p. 314–25.

[28] Liu Y Lin D Wu B Zhou W. Ketamine abuse potential and use disorder. Brain Res Bull (2016) 126:68–73. 10.1016/j.brainresbull.2016.05.016 27261367

[29] Romeo B Choucha W Fossati P Rotge JY. Meta-analysis of short- and mid-term efficacy of ketamine in unipolar and bipolar depression. Psychiatry Res (2015) 230:682–8. 10.1016/j.psychres.2015.10.032 26548981

[30] Müller CP Hassan Z The evaluation of new psychoactive drugs. Brain Res Bull (2016) 126:1–2. 10.1016/j.brainresbull.2016.05.013 27235862

[31] Hassan Z Muzaimi M Navaratnam V Yusoff NHM Suhaimi FW Vadivelu R From Kratom to mitragynine and its derivatives: physiological and behavioural effects related to use, abuse, and addiction. Neurosci Biobehav Rev (2013) 37:138–51. 10.1016/j.neubiorev.2012.11.012 23206666

[32] Singh D Narayanan S Vicknasingam B. Traditional and non-traditional uses of mitragynine (Kratom): a survey of the literature. Brain Res Bull (2016) 126(Pt 1):41–6. 10.1016/j.brainresbull.2016.05.004 27178014

[33] Brown PN Lund JA Murch SJ. A botanical, phytochemical and ethnomedicinal review of the genus *Mitragyna korth*: implications for products sold as Kratom. J Ethnopharmacol (2017) 202:302–25. 10.1016/j.jep.2017.03.020 28330725

[34] Saingam D Assanangkornchai S Geater AF Lerkiatbundit S Validation of Krathom (*Mitragyna speciosa Korth*.) dependence scale (KDS): a dependence screen for internationally emerging psychoactive substance. Subst Abuse (2014) 35:276–83. 10.1080/08897077.2014.924464 24853660

[35] Vicknasingam B Narayanan S Beng GT Mansor SM. The informal use of ketum (*Mitragyna speciosa*) for opioid withdrawal in the northern states of peninsular Malaysia and implications for drug substitution therapy. Int J Drug Policy (2010) 21:283–8. 10.1016/j.drugpo.2009.12.003 20092998

[36] Boyer EW Babu KM Adkins JE McCurdy CR Halpern JH Self-treatment of opioid withdrawal using Kratom (*Mitragyna speciosa Korth*). Addiction (2008) 103:1048–50. 10.1111/j.1360-0443.2008.02209.x 18482427 PMC3670991

[37] Prozialeck WC Jivan JK Andurkar SV. Pharmacology of Kratom: an emerging botanical agent with stimulant, analgesic and opioid-like effects. J Am Osteopath Assoc (2012) 112(12):792–9. 23212430

[38] Cinosi E Martinotti G Simonato P Singh D Demetrovics Z Roman-Urrestarazu A Following “the roots” of Kratom (*Mitragyna speciosa*): the evolution of an enhancer from a traditional use to increase work and productivity in Southeast Asia to a recreational psychoactive drug in western countries. Biomed Res Int (2015) 2015:968786 10.1155/2015/968786 26640804 PMC4657101

[39] Warner ML Kaufman NC Grundmann O. The pharmacology and toxicology of Kratom: from traditional herb to drug of abuse. Int J Legal Med (2016) 130:127–38. 10.1007/s00414-015-1279-y 26511390

[40] Swogger MT Hart E Erowid F Erowid E Trabold N Yee K Experiences of Kratom users: a qualitative analysis. J Psychoactive Drugs (2015) 47(5):360–7. 10.1080/02791072.2015.1096434 26595229 PMC12818578

[41] Suhaimi FW Yusoff NHM Hassan R Mansor SM Navaratnam V Müller CP Neurobiology of Kratom and its main alkaloid mitragynine. Brain Res Bull (2016) 126:29–40. 10.1016/j.brainresbull.2016.03.015 27018165

[42] Ahmad K Aziz Z *Mitragyna speciosa* use in the northern states of Malaysia: cross-sectional study. J Ethnopharmacol (2012) 141:446–50. 10.1016/j.jep.2012.03.009 22440259

[43] Saingam D Assanangkornchai S Geater AF Balthip Q Pattern and consequences of Krathom (*Mitragyna speciosa Korth*.) use among male villagers in southern Thailand: a qualitative study. Int J Drug Policy (2012) 24(4):351–8. 10.1016/j.drugpo.2012.09.004 23083922

[44] Tanguay P Kratom in Thailand: decriminalisation and community control? Legislative Reform of Drug Policies. (Vol. 13) Amsterdam: Transnational Institute (2011). p. 1–16.

[45] Assanangkornchai S Muekthong A Sam-Angsri N Pattanasattayawong U The use of mitragynine species (“Krathom”), an addictive plant, in Thailand. Subst Use Misuse (2007) 42:2145–57. 10.1080/10826080701205869 18097996

[46] Singh D Müller CP Vicknasingam BK Mansor SM. Social functioning of Kratom (*Mitragyna speciosa*) users in Malaysia. J Psychoactive Drugs (2015) 47(2):125–31. 10.1080/02791072.2015.1012610 25950592

[47] Trakulsrichai S Tongpo A Sriapha C Wongvisawakorn S Rittilert P Kaojarern S Kratom abuse in Ramathibodi Poison Center, Thailand. A five-year experience. J Psychoactive Drugs (2013) 45(5):404–8. 10.1080/02791072.2013.844532 24592666

[48] Ward J Rosenbaum C Hernon C McCurdy CR Boyer EW Herbal medicines for the management of opioid addiction. CNS Drugs (2011) 25(12):999–1007. 10.2165/11596830-000000000-00000 22133323

[49] Suwanlert S A study of Kratom eaters in Thailand. Bull Narc (1975) 27(3):21–7. 1041694

[50] Prozialeck WC. Update on the pharmacology and legal status of Kratom. J Am Osteopath Assoc (2016) 116(12):802–9. 10.7556/jaoa.2016.156 27893147

[51] Schmidt MM Sharma A Schifano F Feinmann C “Legal highs” on the net – evaluation of UK-based websites, products and product information. Forensic Sci Int (2011) 206:92–7. 10.1016/j.forsciint.2010.06.030 20650576

[52] Kronstrand R Roman M Thelander G Eriksson A. Unintentional fatal intoxications with mitragynine and O-desmethyltramadol from the herbal blend krypton. J Anal Toxicol (2011) 35:242–7. 10.1093/anatox/35.4.242 21513619

[53] Anwar M Law R Schier J Notes from the field: Kratom (*Mitragyna speciosa*) exposures reported to poison centers – United States, 2010-2015. MMWR Morb Mortal Wkly Rep (2016) 65(29):748–9. 10.15585/mmwr.mm6529a4 27466822

[54] Lydecker AG Sharma A McCurdy CR Avery BA Babu KM Boyer EW. Suspected adulteration of commercial Kratom products with 7-hydroxymitragynine. J Med Toxicol (2016) 12(4):341–9. 10.1007/s13181-016-0588-y 27752985 PMC5135684

[55] Shellard EJ The alkaloids of *Mitragyna* with special reference to those of *M. speciosa korth*. Bull Narc (1974) 26(2):41–55. 4607551

[56] Yusoff NHM Mansor SM Müller CP Hassan Z Opioid receptors mediate the acquisition, but not the expression of mitragynine-induced conditioned place preference in rats. Behav Brain Res (2017) 332:1–6. 10.1016/j.bbr.2017.05.059 28559179

[57] Takayama H Maeda M Ohbayashi S Kitajima M Sakai SI Aimi N The first total synthesis of (−)-mitragynine, an analgesic indole alkaloid in *Mitragyna speciosa*. Tetrahed Lett (1995) 36:9337–40. 10.1016/0040-4039(95)02022-H

[58] Ma J Yin W Zhou H Cook JM Total synthesis of the opioid agonistic indole alkaloid mitragynine and the first total syntheses of 9-methoxygeissoschizol and 9-methoxy-Nb-methylgeissoschizol. Organic Lett (2007) 9:3491–4. 10.1021/ol071220l PMC2526544 17685530

[59] Ma J Yin WY Zhou H Liao XB Cook JM General approach to the total synthesis of 9-methoxy-substituted indole alkaloids: synthesis of mitragynine, as well as 9-methoxygeissoschizol and 9-methoxy-N-b-methylgeissoschizol. J Org Chem (2009) 74:264–73. 10.1021/jo801839t 19046119 PMC2654583

[60] Isabel K Total Synthesis of (−)-Mitragynine and Analogues. Master Thesis, Universiteit van Amsterdam, Amsterdam (2012).

[61] Parthasarathy S Ramanathan S Ismail S Adenan MI Mansor SM Murugaiyah V. Determination of mitragynine in plasma with solid-phase extraction and rapid HPLC-UV analysis, and its application to a pharmacokinetic study in rat. Anal Bioanal Chem (2010) 397:2023–30. 10.1007/s00216-010-3707-7 20454783

[62] Janchawee B Keawpradub N Chittrakarn S Prasettho S Wararatananurak P Sawangjareon K. A high-performance liquid chromatographic method for determination of mitragynine in serum and its application to a pharmacokinetic study in rats. Biomed Chromatogr (2007) 21:176–83. 10.1002/bmc.731 17221920

[63] de Moraes NV Moretti RA Furr EB III McCurdy CR Lanchote VL. Determination of mitragynine in rat plasma by LC-MS/MS: application to pharmacokinetics. J Chromatogr B Analyt Technol Biomed Life Sci (2009) 877:2593–7. 10.1016/j.jchromb.2009.06.023 19589735 PMC9258519

[64] Ramanathan S Parthasarathy S Murugaiyah V Magosso E Tan SC Mansor SM. Understanding the physicochemical properties of mitragynine, a principal alkaloid of *Mitragyna speciosa*, for preclinical evaluation. Molecules (2015) 20:4915–27. 10.3390/molecules20034915 25793541 PMC6272646

[65] Philipp AA Wissenbach DK Zoerntlein SW Klein ON Kanogsunthornrat J Maurer HH. Studies on the metabolism of mitragynine, the main alkaloid of the herbal drug Kratom, in rat and human urine using liquid chromatography-linear ion trap mass spectrometry. J Mass Spectrom (2009) 44:1249–61. 10.1002/jms.1607 19536806

[66] Philipp AA Wissenbach DK Weber AA Zapp J Maurer HH Phase I and II metabolites of speciogynine, a diastereomer of the main Kratom alkaloid mitragynine, identified in rat and human urine by liquid chromatography coupled to low- and high-resolution linear ion trap mass spectrometry. J Mass Spectrom (2010) 45:1344–57. 10.1002/jms.1848 20967737

[67] Philipp AA Wissenbach DK Weber AA Zapp J Zoerntlein SW Kanogsunthornrat J Use of liquid chromatography coupled to low- and high-resolution linear ion trap mass spectrometry for studying the metabolism of paynantheine, an alkaloid of the herbal drug Kratom in rat and human urine. Anal Bioanal Chem (2010) 396:2379–91. 10.1007/s00216-009-3239-1 19902190

[68] Taufik Hidayat M Apryani E Nabishah BM Moklas MAA Sharida F Farhan MA Determination of mitragynine bound opioid receptors. Adv Med Dent Sci (2010) 3:65–70.

[69] Yamamoto LT Horie S Takayama H Aimi N Sakai S Yano S Opioid receptor agonistic characteristics of mitragynine pseudoindoxyl in comparison with mitragynine derived from Thai medicinal plant *Mitragyna speciosa*. Gen Pharmacol (1999) 33:73–81. 10.1016/S0306-3623(98)00265-1 10428019

[70] Watanabe K Yano S Horie S Yamamoto LT. Inhibitory effect of mitragynine, an alkaloid with analgesic effect from Thai medicinal plant *Mitragyna speciosa*, on electrically stimulated contraction of isolated guinea-pig ileum through the opioid receptor. Life Sci (1997) 60:933–42. 10.1016/S0024-3205(97)00023-4 9061050

[71] Shamima AR Fakurazi S Hidayat MT Hairuszah I Moklas MA Arulselvan P. Antinociceptive action of isolated mitragynine from *Mitragyna speciosa* through activation of opioid receptor system. Int J Mol Sci (2012) 13:11427–42. 10.3390/ijms130911427 23109863 PMC3472755

[72] Matsumoto K Yamamoto LT Watanabe K Yano S Shan J Pang PK Inhibitory effect of mitragynine, an analgesic alkaloid from Thai herbal medicine, on neurogenic contraction of the vas deferens. Life Sci (2005) 78:187–94. 10.1016/j.lfs.2005.04.042 16107269

[73] Tohda M Thongpraditchote S Matsumoto K Murakami Y Sakai S Aimi N Effects of mitragynine on cAMP formation mediated by delta-opiate receptors in NG108-15 cells. Biol Pharm Bull (1997) 20:338–40. 10.1248/bpb.20.338 9145205

[74] Jamil MF Subki MF Lan TM Majid MI Adenan MI. The effect of mitragynine on cAMP formation and mRNA expression of mu-opioid receptors mediated by chronic morphine treatment in SK-N-SH neuroblastoma cell. J Ethnopharmacol (2013) 148:135–43. 10.1016/j.jep.2013.03.078 23608241

[75] Fakurazi S Rahman SA Hidayat MT Ithnin H Moklas MA Arulselvan P. The combination of mitragynine and morphine prevents the development of morphine tolerance in mice. Molecules (2013) 18:666–81. 10.3390/molecules18010666 23292329 PMC6270191

[76] Reanmongkol W Keawpradub N Sawangjaroen K Effects of the extracts from *Mitragyna speciosa Korth*. Leaves on analgesic and behavioral activities in experimental animals. Songkl J Sci Technol (2007) 29:39–48.

[77] Sabetghadam A Ramanathan S Mansor SM. The evaluation of antinociceptive activity of alkaloid, methanolic, and aqueous extracts of Malaysian *Mitragyna speciosa Korth* leaves in rats. Pharmacognosy Res (2010) 2:181–5. 10.4103/0974-8490.65514 21808563 PMC3141311

[78] Shaik Mossadeq WM Sulaiman MR Tengku Mohamad TA Chiong HS Zakaria ZA Jabit ML Anti-inflammatory and antinociceptive effects of *Mitragyna speciosa Korth* methanolic extract. Med Princ Pract (2009) 18:378–84. 10.1159/000226292 19648761

[79] Criddle CA A Comparison of Mitragyna speciosa and Mitragynine against Opioids on Thermal Nociception in Rats. (2015). Available from: http://thesis.honors.olemiss.edu 10.1016/j.fitote.2015.12.001 26688378

[80] Carpenter JM Criddle CA Craig HK Ali Z Zhang Z Khan IA Comparative effects of *Mitragyna speciosa* extract, mitragynine, and opioid agonists on thermal nociception in rats. Fitoterapia (2016) 109:87–90. 10.1016/j.fitote.2015.12.001 26688378

[81] Farah Idayu N Hidayat MT Moklas MA Sharida F Raudzah AR Shamima AR Antidepressant-like effect of mitragynine isolated from *Mitragyna speciosa Korth* in mice model of depression. Phytomedicine (2011) 18:402–7. 10.1016/j.phymed.2010.08.011 20869223

[82] Kumarnsit E Vongvatcharanon U Keawpradub N Intasaro P. Fos-like immunoreactivity in rat dorsal raphe nuclei induced by alkaloid extract of *Mitragyna speciosa*. Neurosci Lett (2007) 416:128–32. 10.1016/j.neulet.2007.01.061 17316993

[83] Saitoh A Yamada M. Antidepressant-like effects of δ opioid receptor agonists in animal models. Curr Neuropharmacol (2012) 10:231–8. 10.2174/157015912803217314 23449756 PMC3468877

[84] Yusoff NHM Suhaimi FW Vadivelu RK Hassan Z Rümler A Rotter A Abuse potential and adverse cognitive effects of mitragynine (Kratom). Addict Biol (2016) 21:98–110. 10.1111/adb.12185 25262913

[85] Apryani E Hidayat MT Moklas MA Fakurazi S Idayu NF. Effects of mitragynine from *Mitragyna speciosa Korth* leaves on working memory. J Ethnopharmacol (2010) 129:357–60. 10.1016/j.jep.2010.03.036 20371280

[86] Hazim AI Mustapha M Mansor SM The effects on motor behaviour and short-term memory tasks in mice following an acute administration of *Mitragyna speciosa* alkaloid extract and mitragynine. J Med Plants Res (2011) 5:5810–7.

[87] Senik MH Mansor SM John Tharakan KJ Abdullah JM Effect of acute administration of *Mitragyna speciosa Korth*. Standardized methanol extract in animal model of learning and memory. J Med Plants Res (2012) 6:1007–14. 10.5897/JMPR11.601

[88] Ismail NIW Jayabalan N Mansor SM Müller CP Muzaimi M. Chronic mitragynine (Kratom) enhances punishment resistance in natural reward seeking and impairs place learning in mice. Addict Biol (2017) 22:967–76. 10.1111/adb.12385 26990882

[89] Harun N Hassan Z Navaratnam V Mansor SM Shoaib M. Discriminative stimulus properties of mitragynine (Kratom) in rats. Psychopharmacology (2015) 232:2227–38. 10.1007/s00213-015-3866-5 25616583

[90] Davies S Wood DM Smith G Button J Ramsey J Archer R Purchasing ‘legal highs’ on the Internet—is there consistency in what you get? QJM (2010) 103(7):489–93. 10.1093/qjmed/hcq056 20413562

[91] Kemp A Clark M Dobbs T Galli R Sherman J Cox R Top 10 facts you should know. Synthetic cannabinoids: not so nice spice. Am J Med (2016) 129(3):240–4.e1. 10.1016/j.amjmed.2015.10.008 26522795

[92] WHO. Expert committee on drug dependence. Thirty-sixth report. World Health Organ Tech Rep Ser (2015) 991:1–50. 26062388

[93] Hall W Degenhardt L High potency cannabis: a risk factor for dependence, poor psychosocial outcomes, and psychosis. Br Med J (2015) 350:h1205 10.1136/bmj.h1205 25739398

[94] Torjesen I High potency cannabis is associated with tripled risk of psychosis, study indicates. Br Med J (2015) 350:h939 10.1136/bmj.h939 25694180

[95] Di Forti M Morgan C Dazzan P Pariante C Mondelli V Marques TR High-potency cannabis and the risk of psychosis. Br J Psychiatry (2009) 195(6):488–91. 10.1192/bjp.bp.109.064220 19949195 PMC2801827

[96] Di Forti M Sallis H Allegri F Trotta A Ferraro L Stilo SA Daily use, especially of high-potency cannabis, drives the earlier onset of psychosis in cannabis users. Schizophr Bull (2014) 40(6):1509–17. 10.1093/schbul/sbt181 24345517 PMC4193693

[97] Forrester MB Kleinschmidt K Schwarz E Young A. Synthetic cannabinoid and marijuana exposures reported to poison centers. Hum Exp Toxicol (2012) 31(10):1006–11. 10.1177/0960327111421945 22859662

[98] Debruyne D Le Boisselier R. Emerging drugs of abuse: current perspectives on synthetic cannabinoids. Subst Abuse Rehabil (2015) 6:113–29. 10.2147/SAR.S73586 26543389 PMC4622447

[99] Barratt MJ Potter GR Wouters M Wilkins C Werse B Perälä J Lessons from conducting trans-national Internet-mediated participatory research with hidden populations of cannabis cultivators. Int J Drug Policy (2015) 26(3):238–49. 10.1016/j.drugpo.2014.12.004 25576247

[100] Gurdal F Asirdizer M Aker RG Korkut S Gocer Y Kucukibrahimoglu EE Review of detection frequency and type of synthetic cannabinoids in herbal compounds analyzed by Istanbul Narcotic Department of the Council of Forensic Medicine, Turkey. J Forensic Leg Med (2013) 20(6):667–72. 10.1016/j.jflm.2013.03.041 23910858

[101] Mills B Yepes A Nugent K. Synthetic cannabinoids. Am J Med Sci (2015) 350(1):59–62. 10.1097/MAJ.0000000000000466 26132518

[102] Kadaria D Sinclair SE A case of acute agitation with a negative urine drug screen: a new wave of legal drugs of abuse. Tenn Med (2012) 105(9):31–2. 23097956

[103] Weaver MF Hopper JA Gunderson EW. Designer drugs 2015: assessment and management. Addict Sci Clin Pract (2015) 10:8. 10.1186/s13722-015-0024-7 25928069 PMC4422150

[104] Khullar V Jain A Sattari M. Emergence of new classes of recreational drugs – synthetic cannabinoids and cathinones. J Gen Intern Med (2014) 29(8):1200–4. 10.1007/s11606-014-2802-4 24553958 PMC4099455

[105] Forrester MB Kleinschmidt K Schwarz E Young A. Synthetic cannabinoid exposures reported to Texas poison centers. J Addict Dis (2011) 30(4):351–8. 10.1080/10550887.2011.609807 22026527

[106] Müller H Huttner HB Köhrmann M Wielopolski JE Kornhuber J Sperling W Panic attack after spice abuse in a patient with ADHD. Pharmacopsychiatry (2010) 43(4):152–3. 10.1055/s-0029-1243252 20127596

[107] Müller H Sperling W Köhrmann M Huttner HB Kornhuber J Maler JM The synthetic cannabinoid spice as a trigger for an acute exacerbation of cannabis induced recurrent psychotic episodes. Schizophr Res (2010) 118(1–3):309–10. 10.1016/j.schres.2009.12.001 20056392

[108] Loeffler G Delaney E Hann M. International trends in spice use: prevalence, motivation for use, relationship to other substances, and perception of use and safety for synthetic cannabinoids. Brain Res Bull (2016) 126(Pt 1):8–28. 10.1016/j.brainresbull.2016.04.013 27108542

[109] Castaneto MS Gorelick DA Desrosiers NA Hartman RL Pirard S Huestis MA. Synthetic cannabinoids: epidemiology pharmacodynamics, and clinical implications. Drug Alcohol Depend (2014) 144:12–41. 10.1016/j.drugalcdep.2014.08.005 25220897 PMC4253059

[110] Seely KA Lapoint J Moran JH Fattore L. Spice drugs are more than harmless herbal blends: a review of the pharmacology and toxicology of synthetic cannabinoids. Prog Neuropsychopharmacol Biol Psychiatry (2012) 39(2):234–43. 10.1016/j.pnpbp.2012.04.017 22561602 PMC3936256

[111] Trecki J Gerona RR Schwartz MD Synthetic cannabinoid-related illnesses and deaths. N Engl J Med (2015) 373:103–7. 10.1056/NEJMp1505328 26154784

[112] Wiley JL Marusich JA Martin BR Huffman JW. 1-Pentyl-3-phenylacetylindoles and JWH-018 share in vivo cannabinoid profiles in mice. Drug Alcohol Depend (2012) 123(1–3):148–53. 10.1016/j.drugalcdep.2011.11.001 22127210 PMC3294131

[113] Ernst L Schiebel HM Theuring C Lindigkeit R Beuerle T Identification and characterization of JWH-122 used as new ingredient in spice-like herbal incenses. Forensic Sci Int (2011) 208(1–3):e31–5. 10.1016/j.forsciint.2011.03.020 21482054

[114] Musshoff F Hottmann L Hess C Madea B Legal highs from the German Internet—bath salt drugs on the rise. Arch Kriminol (2013) 232(3–4):91–103. 24358620

[115] Hillebrand J Olszewski D Sedefov R. Legal highs on the Internet. Subst Use Misuse (2010) 45(3):330–40. 10.3109/10826080903443628 20141450

[116] Möller I Wintermeyer A Bender K Jübner M Thomas A Krug O Screening for the synthetic cannabinoid JWH-018 and its major metabolites in human doping controls. Drug Test Anal (2011) 3(9):609–20. 10.1002/dta.158 20872894

[117] Strano-Rossi S Anzillotti L Castrignanò E Romolo FS Chiarotti M Ultra high performance liquid chromatography–electrospray ionization-tandem mass spectrometry screening method for direct analysis of designer drugs, spice and stimulants in oral fluid. J Chromatogr A (2012) 1258:37–42. 10.1016/j.chroma.2012.07.098 22939380

[118] Hudson S Ramsey J. The emergence and analysis of synthetic cannabinoids. Drug Test Anal (2011) 3(7–8):466–78. 10.1002/dta.268 21337724

[119] Sundström M Pelander A Angerer V Hutter M Kneisel S Ojanperä I A high-sensitivity ultra-high performance liquid chromatography/high-resolution time-of-flight mass spectrometry (UHPLC–HR–TOFMS) method for screening synthetic cannabinoids and other drugs of abuse in urine. Anal Bioanal Chem (2013) 405(26):8463–74. 10.1007/s00216-013-7272-8 23954996

[120] Grabenauer M Krol WL Wiley JL Thomas BF. Analysis of synthetic cannabinoids using high-resolution mass spectrometry and mass defect filtering: implications for non targeted screening of designer drugs. Anal Chem (2012) 84(13):5574–81. 10.1021/ac300509h 22724537 PMC3400111

[121] Muller-Vahl K Grotenhermen F Cannabis therapy. Dtsch Arztebl Int (2013) 110(9):144 10.3238/arztebl.2013.0144a PMC3601285 23533555

[122] Grotenhermen F. Pharmacology of cannabinoids. Neuro Endocrinol Lett (2004) 25(1–2):14–23. 15159677

[123] Grotenhermen F. Cannabinoids. Curr Drug Targets CNS Neurol Disord (2005) 4(5):507–30. 10.2174/156800705774322111 16266285

[124] Gunderson EW Synthetic cannabinoids: a new frontier of designer drugs. Ann Intern Med (2013) 159(8):563–4. 10.7326/0003-4819-159-8-201310150-00707 24018791

[125] Rosenbaum CD Carreiro SP Babu KM Here today, gone tomorrow and back again? A review of herbal marijuana alternatives (K2, spice), synthetic cathinones (bath salts), Kratom, *Salvia divinorum*, methoxetamine, and piperazines. J Med Toxicol (2012) 8(1):15–32. 10.1007/s13181-011-0202-2 22271566 PMC3550220

[126] Penders T Saeed S Synthetic cannabinoids and bath salts should be considered drugs of abuse. Am Fam Physician (2012) 85(9):852. 22612177

[127] McGuinness TM Newell D. Risky recreation: synthetic cannabinoids have dangerous effects. J Psychosoc Nurs Ment Health Serv (2012) 50(8):16–8. 10.3928/02793695-20120703-04 22801822

[128] Bhanushali GK Jain G Fatima H Leisch LJ Thornley-Brown D AKI associated with synthetic cannabinoids: a case series. Clin J Am Soc Nephrol (2013) 8(4):523–6. 10.2215/CJN.05690612 23243266 PMC3613952

[129] Van der Veer N Friday J Persistent psychosis following the use of spice. Schizophr Res (2011) 130(1–3):285–6. 10.1016/j.schres.2011.04.022 21602030

[130] Brakoulias V Products containing synthetic cannabinoids and psychosis. Aust N Z J Psychiatry (2012) 46(3):281–2. 10.1177/0004867411433974 22391292

[131] Jinwala FN Gupta M. Synthetic cannabis and respiratory depression. J Child Adolesc Psychopharmacol (2012) 22(6):459–62. 10.1089/cap.2011.0122 23234589

[132] Harris CR Brown A. Synthetic cannabinoid intoxication: a case series and review. J Emerg Med (2013) 44(2):360–6. 10.1016/j.jemermed.2012.07.061 22989695

[133] Oluwabusi OO Lobach L Akhtar U Youngman B Ambrosini PJ Synthetic cannabinoid-induced psychosis: two adolescent cases. J Child Adolesc Psychopharmacol (2012) 22(5):393–5. 10.1089/cap.2012.0004 23083027

[134] Goshgarian AM Benford DM Caplan JP Bath salt abuse: neuropsychiatric effects of cathinone derivatives. Psychosomatics (2011) 52(6):593–4. 10.1016/j.psym.2011.03.003 22054633

[135] Benford DM Caplan JP Psychiatric sequelae of spice, K2, and synthetic cannabinoid receptor agonists. Psychosomatics (2011) 52(3):295 10.1016/j.psym.2011.01.004 21565605

[136] Forrester MB Haywood T. Geographic distribution of synthetic cannabinoid exposures reported to Texas poison centers. Am J Drug Alcohol Abuse (2012) 38(6):603–8. 10.3109/00952990.2012.670339 22571605

[137] Forrester MB. Adolescent synthetic cannabinoid exposures reported to Texas poison centers. Pediatr Emerg Care (2012) 28(10):985–9. 10.1097/PEC.0b013e31826c9a97 23023462

[138] Durand D Delgado LL de la Parra-Pellot DM Nichols-Vinueza D. Psychosis and severe rhabdomyolysis associated with synthetic cannabinoid use: a case report. Clin Schizophr Relat Psychoses (2015) 8(4):205–8. 10.3371/CSRP.DUDE.031513 23518784

[139] Hurst D Loeffler G McLay R Psychosis associated with synthetic cannabinoid agonists: a case series. Am J Psychiatry (2011) 168(10):1119 10.1176/appi.ajp.2011.11010176 21969050

[140] Marshell R Kearney-Ramos T Brents LK Hyatt WS Tai S Prather PL In vivo effects of synthetic cannabinoids JWH-018 and JWH-073 and phytocannabinoid delta9-THC in mice: inhalation versus intraperitoneal injection. Pharmacol Biochem Behav (2014) 124:40–7. 10.1016/j.pbb.2014.05.010 24857780 PMC4340656

[141] Brents LK Prather PL. The K2/spice phenomenon: emergence, identification, legislation and metabolic characterization of synthetic cannabinoids in herbal incense products. Drug Metab Rev (2014) 46(1):72–85. 10.3109/03602532.2013.839700 24063277 PMC4100246

[142] Auwärter V Dresen S Weinmann W Müller M Pütz M Ferreirós N ‘Spice’ and other herbal blends: harmless incense or cannabinoid designer drugs? J Mass Spectrom (2009) 44(5):832–7. 10.1002/jms.1558 19189348

[143] Grigoryev A Savchuk S Melnik A Moskaleva N Dzhurko J Ershov M Chromatography–mass spectrometry studies on the metabolism of synthetic cannabinoids JWH-018 and JWH-073, psychoactive components of smoking mixtures. J Chromatogr B Analyt Technol Biomed Life Sci (2011) 879(15–16):1126–36. 10.1016/j.jchromb.2011.07.004 21481654

[144] Grigoryev A Kavanagh P Melnik A The detection of the urinary metabolites of 3-[(adamantan-1-yl) carbonyl]-1-pentylindole (AB-001), a novel cannabimimetic, by gas chromatography–mass spectrometry. Drug Test Anal (2012) 4(6):519–24. 10.1002/dta.350 22102533

[145] Grigoryev A Kavanagh P Melnik A The detection of the urinary metabolites of 1-[(5-fluoropentyl)-1H-indol-3-yl]-(2-iodophenyl) methanone(AM-694): a high affinity cannabimimetic, by gas chromatography–mass spectrometry. Drug Test Anal (2013) 5(2):110–5. 10.1002/dta.1336 22522907

[146] Ortega A Rangel-López E Hidalgo-Miranda A Morales A Ruiz-García E Meneses-García A On the effects of CP 55-940 and other cannabinoid receptor agonists in C6 and U373 cell lines. Toxicol In Vitro (2015) 29(7):1941–51. 10.1016/j.tiv.2015.08.003 26255146

[147] Latorre JG Schmidt EB. Cannabis, cannabinoids, and cerebral metabolism: potential applications in stroke and disorders of the central nervous system. Curr Cardiol Rep (2015) 17(9):627. 10.1007/s11886-015-0627-3 26238742

[148] Soderstrom K Tian Q CB(1) cannabinoid receptor activation dose dependently modulates neuronal activity within caudal but not rostral song control regions of adult Zebra finch telencephalon. Psychopharmacology (Berl) (2008) 199(2):265–73. 10.1007/s00213-008-1190-z 18509622 PMC2586593

[149] Cui YY D’Agostino B Risse PA Marrocco G Naline E Zhang Y Cannabinoid CB(2) receptor activation prevents bronchoconstriction and airway oedema in a model of gastro-oesophageal reflux. Eur J Pharmacol (2007) 573(1–3):206–13. 10.1016/j.ejphar.2007.06.040 17643417

[150] Cox ML Haller VL Welch SP. The antinociceptive effect of delta9-tetrahydrocannabinol in the arthritic rat involves the CB(2)cannabinoid receptor. Eur J Pharmacol (2007) 570(1–3):50–6. 10.1016/j.ejphar.2007.05.024 17588560

[151] Darmani NA. Delta(9)-tetrahydrocannabinol and synthetic cannabinoids prevent emesis produced by the cannabinoid CB(1) receptor antagonist/inverse agonist SR 141716A. Neuropsychopharmacology (2001) 24(2):198–203. 10.1016/S0893-133X(00)00197-4 11120402

[152] Acquas E Pisanu A Marrocu P Di Chiara G. Cannabinoid CB(1) receptor agonists increase rat cortical and hippocampal acetylcholine release in vivo. Eur J Pharmacol (2000) 401(2):179–85. 10.1016/S0014-2999(00)00403-9 10924924

[153] Buckley NE McCoy KL Mezey E Bonner T Zimmer A Felder CC Immunomodulation by cannabinoids is absent in mice deficient for the cannabinoid CB(2) receptor. Eur J Pharmacol (2000) 396(2–3):141–9. 10.1016/S0014-2999(00)00211-9 10822068

[154] Underwood E Alarm over synthetic cannabinoids. Science (2015) 347(6221):473 10.1126/science.347.6221.473 25635070

[155] Carbonaro TM Gatch MB. Neuropharmacology of *N,N*-dimethyltryptamine. Brain Res Bull (2016) 126:74–88. 10.1016/j.brainresbull.2016.04.016 27126737 PMC5048497

[156] Strassman R DMT: The Spirit Molecule. A Doctor’s Revolutionary Research into the Biology of Near Death and Mystical Experiences. United States: Park Street Press (2001).

[157] Callaway JC Raymon LP Hearn WL McKenna DJ Grob CS Brito GS Quantitation of *N,N*-dimethyltryptamine and harmala alkaloids in human plasma after oral dosing with ayahuasca. J Anal Toxicol (1996) 20:492–7. 10.1093/jat/20.6.492 8889686

[158] Ott J. Pharmahuasca: human pharmacology of oral DMT plus harmine. J Psychoactive Drugs (1999) 31(2):171–7. 10.1080/02791072.1999.10471741 10438001

[159] Wallach JV. Endogenous hallucinogens as ligands of the trace amine receptors: a possible role in sensory perception. Med Hypotheses (2009) 72:91–4. 10.1016/j.mehy.2008.07.052 18805646

[160] Barker SA Borjigin J Lomnicka I Strassman R LC/MS/MS analysis of the endogenous dimethyltryptamine hallucinogens, their precursors and major metabolites in rat pineal gland microdialysate. Biomed Chromatogr (2013) 27:1690–700. 10.1002/bmc.2981 23881860

[161] Christian ST Harrison R Quayle E Pagel J Monti J The in vitro identification of dimethyltryptamine (DMT) in mammalian brain and its characterization as a possible endogenous neuroregulatory agent. Biochem Med (1977) 18:164–83. 10.1016/0006-2944(77)90088-6 20877

[162] Keiser MJ Setola V Irwin JJ Laggner C Abbas AI Hufeisen SJ Predicting new molecular targets for known drugs. Nature (2009) 462:175–81. 10.1038/nature08506 19881490 PMC2784146

[163] McKenna DJ. Clinical investigations of the therapeutic potential of ayahuasca: rationale and regulatory challenges. Pharmacol Ther (2004) 102:111–29. 10.1016/j.pharmthera.2004.03.002 15163593

[164] Dos Santos RG Valle M Bouso JC Nomdedéu JF Rodríguez-Espinosa J McIlhenny EH Autonomic, neuroendocrine, and immunological effects of ayahuasca: a comparative study with d-amphetamine. J Clin Psychopharmacol (2011) 31:717–26. 10.1097/JCP.0b013e31823607f6 22005052

[165] Dos Santos RG Grasa E Valle M Ballester MR Bouso JC Nomdedéu JF Pharmacology of ayahuasca administered in two repeated doses. Psychopharmacology (2012) 219:1039–53. 10.1007/s00213-011-2434-x 21842159

[166] Halberstadt AL Nichols DE Serotonin and serotonin receptors in hallucinogen action. In: Müller CP Jacobs BL, editors. Handbook of the Behavioral Neurobiology of Serotonin. London: Academic Press (2010). p. 621–36.

[167] Carbonaro TM Eshleman AJ Forster MJ Cheng K Rice KC Gatch MB. The role of 5-HT2A, 5-HT 2C and mGlu2 receptors in the behavioral effects of tryptamine hallucinogens *N,N-*dimethyltryptamine and N,N-diisopropyltryptamine in rats and mice. Psychopharmacology (2015) 232:275–84. 10.1007/s00213-014-3658-3 24985890 PMC4282596

[168] Cole JM Pieper WA The effects of *N,N-*dimethyltryptamine on operant behaviour in squirrel monkeys. Psychopharmacology (1973) 29:107–12. 10.1007/BF00422642 4196867

[169] Strassman RJ Qualls CR Berg LM. Differential tolerance to biological and subjective effects of four closely spaced doses of *N,N*-dimethyltryptamine in humans. Biol Psychiatry (1996) 39:784–95. 10.1016/0006-3223(95)00200-6 8731519

[170] Glennon RA Young R Jacyno JM Slusher M Rosecrans JA. DOM-stimulus generalization to LSD and other hallucinogenic indolealkylamines. Eur J Pharmacol (1983) 86:453–9. 10.1016/0014-2999(83)90196-6 6572591

[171] Glennon RA. Discriminative stimulus properties of phenylisopropylamine derivatives. Drug Alcohol Depend (1986) 17:119–34. 10.1016/0376-8716(86)90003-7 2874967

[172] Riba J Rodríguez-Fornells A Urbano G Morte A Antonijoan R Montero M Subjective effects and tolerability of the South American psychoactive beverage ayahuasca in healthy volunteers. Psychopharmacology (2001) 154:85–95. 10.1007/s002130000606 11292011

[173] Frecska E Ayahuasca versus violence – a case report. Neuropsychopharmacol Hung (2008) 10(2):103–6. 18959142

[174] Frecska E Bokor P Winkelman M. The therapeutic potentials of ayahuasca: possible effects against various diseases of civilization. Front Pharmacol (2016) 7:35. 10.3389/fphar.2016.00035 26973523 PMC4773875

[175] Szabo A Frecska E Dimethyltrypt-amine (DMT): a biochemical Swiss Army knife in neuroinflammation and neuroprotection? Neural Regen Res (2016) 11(3):396–7. 10.4103/1673-5374.179041 27127466 PMC4828992

[176] Pal A Fontanilla D Gopalakrishnan A Chae YK Markley JL Ruoho AE. The sigma-1 receptor protects against cellular oxidative stress and activates antioxidant response elements. Eur J Pharmacol (2012) 682:12–20. 10.1016/j.ejphar.2012.01.030 22381068 PMC3314091

[177] Frecska E Szabo A Winkelman MJ Luna LE McKenna DJ A possibly sigma-1 receptor mediated role of dimethyltryptamine in tissue protection, regeneration, and immunity. J Neural Transm (2013) 120:1295–303. 10.1007/s00702-013-1024-y 23619992

[178] Quednow BB Geyer MA Halberstadt AL Serotonin and schizophrenia. In: Müller CP Jacobs BL, editors. Handbook of the Behavioral Neurobiology of Serotonin. London: Academic Press (2010). p. 585–620.

[179] Checkley SA Murray RM Oon MC Rodnight R Birley JL A longitudinal study of urinary excretion of *N, N*-dimethyltryptamine in psychotic patients. Br J Psychiatry (1980) 137:236–9. 10.1192/bjp.137.3.236 6777009

[180] Osório Fde L Sanches RF Macedo LR Santos RG Maia-de-Oliveira JP Wichert-Ana L Antidepressant effects of a single dose of ayahuasca in patients with recurrent depression: a preliminary report. Rev Bras Psiquiatr (2015) 37:13–20. 10.1590/1516-4446-2014-1496 25806551

[181] Jacob MS Presti DE. Endogenous psychoactive tryptamines reconsidered: an anxiolytic role for dimethyltryptamine. Med Hypotheses (2005) 64:930–7. 10.1016/j.mehy.2004.11.005 15780487

[182] Schenberg EE. Ayahuasca and cancer treatment. SAGE Open Med (2013) 1:1–12. 10.1177/2050312113508389 26770688 PMC4687784

[183] Derocq JM Bourrié B Ségui M Le Fur G Casellas P In vivo inhibition of endotoxin-induced pro-inflammatory cytokines production by the sigma ligand SR-31747. J Pharmacol Exp Ther (1995) 272:224–30. 7815336

[184] Metzner R. Hallucinogenic drugs and plants in psychotherapy and shamanism. J Psychoactive Drugs (1998) 30(4):333–41. 10.1080/02791072.1998.10399709 9924839

[185] El-Mallakh RS Halpern JH Abraham HD Substance abuse: hallucinogen- and MDMA-related disorders. 4th ed In: Tasman A Kay J Lieberman JA First MB Riba M, editors. Psychiatry. (Chap. 76), Hoboken, NJ: Wiley-Blackwell (2015). p. 1480–506.

[186] Nichols DE. Hallucinogens. Pharmacol Ther (2004) 101(2):131–81. 10.1016/j.pharmthera.2003.11.002 14761703

[187] Vollenweider FX Kometer M. The neurobiology of psychedelic drugs: implications for the treatment of mood disorders. Nat Rev Neurosci (2010) 11(9):642–51. 10.1038/nrn2884 20717121

[188] Hollister LE Effects of hallucinogens in humans. In: Jacobs BL, editor. Hallucinogens: Neurochemical, Behavioral, and Clinical Perspectives. New York: Raven Press (1984). p. 19–33.

[189] Nichols DE. Psychedelics. Pharmacol Rev (2016) 68(2):264–355. 10.1124/pr.115.011478 26841800 PMC4813425

[190] Fantegrossi WE Murnane KS Reissig CJ. The behavioral pharmacology of hallucinogens. Biochem Pharmacol (2008) 75(1):17–33. 10.1016/j.bcp.2007.07.018 17977517 PMC2247373

[191] Halberstadt AL Geyer MA. Multiple receptors contribute to the behavioral effects of indoleamine hallucinogens. Neuropharmacology (2011) 61(3):364–81. 10.1016/j.neuropharm.2011.01.017 21256140 PMC3110631

[192] Preller KH Herdener M Pokorny T Planzer A Kraehenmann R Stampfli P The fabric of meaning and subjective effects in LSD-induced states depend on serotonin 2A receptor activation. Curr Biol (2017) 27(3):451–7. 10.1016/j.cub.2016.12.030 28132813

[193] Quednow BB Kometer M Geyer MA Vollenweider FX. Psilocybin-induced deficits in automatic and controlled inhibition are attenuated by ketanserin in healthy human volunteers. Neuropsychopharmacology (2012) 37(3):630–40. 10.1038/npp.2011.228 21956447 PMC3260978

[194] Vollenweider FX Vollenweider-Scherpenhuyzen MF Babler A Vogel H Hell D. Psilocybin induces schizophrenia-like psychosis in humans via a serotonin-2 agonist action. Neuroreport (1998) 9(17):3897–902. 10.1097/00001756-199812010-00024 9875725

[195] Rickli A Luethi D Reinisch J Buchy D Hoener MC Liechti ME Receptor interaction profiles of novel N-2-methoxybenzyl (NBOMe) derivatives of 2,5-dimethoxy-substituted phenethylamines (2C drugs). Neuropharmacology (2015) 99:546–53. 10.1016/j.neuropharm.2015.08.034 26318099

[196] Gonzalez-Maeso J Ang RL Yuen T Chan P Weisstaub NV Lopez-Gimenez JF Identification of a serotonin/glutamate receptor complex implicated in psychosis. Nature (2008) 452(7183):93–7. 10.1038/nature06612 18297054 PMC2743172

[197] Delille HK Mezler M Marek GJ The two faces of the pharmacological interaction of mGlu2 and 5-HT(2)A – relevance of receptor heterocomplexes and interaction through functional brain pathways. Neuropharmacology (2013) 70:296–305. 10.1016/j.neuropharm.2013.02.005 23466331

[198] Hofmann A Psychotomimetic agents. In: Burger A, editor. Chemical Constitution and Pharmacodynamic Action. (Vol. 2), New York: M. Dekker (1968). p. 169–235.

[199] Shulgin AT Shulgin A PIHKAL: A Chemical Love Story. Berkeley: Transform Pres (1991).

[200] Shulgin AT Shulgin A TIHKAL: The Continuation. Berkeley: Transform Pres (1997).

[201] Dean BV Stellpflug SJ Burnett AM Engebretsen KM. 2C or not 2C: phenethylamine designer drug review. J Med Toxicol (2013) 9(2):172–8. 10.1007/s13181-013-0295-x 23494844 PMC3657019

[202] Beharry S Gibbons S. An overview of emerging and new psychoactive substances in the United Kingdom. Forensic Sci Int (2016) 267:25–34. 10.1016/j.forsciint.2016.08.013 27552699

[203] Liechti M. Novel psychoactive substances (designer drugs): overview and pharmacology of modulators of monoamine signaling. Swiss Med Wkly (2015) 145:w14043. 10.4414/smw.2015.14043 25588018

[204] Zawilska JB Andrzejczak D. Next generation of novel psychoactive substances on the horizon – a complex problem to face. Drug Alcohol Depend (2015) 157:1–17. 10.1016/j.drugalcdep.2015.09.030 26482089

[205] Schifano F Papanti GD Orsolini L Corkery JM. Novel psychoactive substances: the pharmacology of stimulants and hallucinogens. Expert Rev Clin Pharmacol (2016) 9(7):943–54. 10.1586/17512433.2016.1167597 26985969

[206] Lawn W Barratt M Williams M Horne A Winstock A. The NBOMe hallucinogenic drug series: patterns of use, characteristics of users and self-reported effects in a large international sample. J Psychopharmacol (2014) 28(8):780–8. 10.1177/0269881114523866 24569095

[207] Simmler LD Rickli A Schramm Y Hoener MC Liechti ME. Pharmacological profiles of aminoindanes, piperazines, and pipradrol derivatives. Biochem Pharmacol (2014) 88(2):237–44. 10.1016/j.bcp.2014.01.024 24486525

[208] Palamar JJ Martins SS Su MK Ompad DC Self-reported use of novel psychoactive substances in a US nationally representative survey: prevalence, correlates, and a call for new survey methods to prevent underreporting. Drug Alcohol Depend (2015) 156:112–9. 10.1016/j.drugalcdep.2015.08.028 26377051 PMC4633323

[209] Vallersnes OM Dines AM Wood DM Yates C Heyerdahl F Hovda KE Psychosis associated with acute recreational drug toxicity: a European case series. BMC Psychiatry (2016) 16:293. 10.1186/s12888-016-1002-7 27538886 PMC4990880

[210] Logan BK Mohr ALA Friscia M Krotulski AJ Papsun DM Kacinko SL Reports of adverse events associated with use of novel psychoactive substances, 2013-2016: a review. J Anal Toxicol (2017):1–38. 10.1093/jat/bkx031 28459969

[211] Burish MJ Thoren KL Madou M Toossi S Shah M. Hallucinogens causing seizures? A case report of the synthetic amphetamine 2,5-dimethoxy-4-chloroamphetamine. Neurohospitalist (2015) 5(1):32–4. 10.1177/1941874414528939 25553227 PMC4272348

[212] Halberstadt AL Pharmacology and toxicology of N-benzylphenethylamine (“NBOMe”) hallucinogens. Curr Top Behav Neurosci (2017) 32:283–311. 10.1007/7854_2016_64 28097528

[213] Hieger MA Rose SR Cumpston KL Stromberg PE Miller S Wills BK. Severe poisoning after self-reported use of 2-(4-iodo-2,5-dimethoxyphenyl)-N-[(2-methoxyphenyl)methyl]ethanamine, a novel substituted amphetamine: a case series. Am J Emerg Med (2015) 33(12):1843.e1–3. 10.1016/j.ajem.2015.04.065 25983267

[214] Srisuma S Bronstein AC Hoyte CO. NBOMe and 2C substitute phenylethylamine exposures reported to the National Poison Data System. Clin Toxicol (Phila) (2015) 53(7):624–8. 10.3109/15563650.2015.1054502 26065360

[215] Baggott MJ Coyle JR Erowid E Erowid F Robertson LC. Abnormal visual experiences in individuals with histories of hallucinogen use: a web-based questionnaire. Drug Alcohol Depend (2011) 114(1):61–7. 10.1016/j.drugalcdep.2010.09.006 21035275

[216] Litjens RP Brunt TM Alderliefste GJ Westerink RH. Hallucinogen persisting perception disorder and the serotonergic system: a comprehensive review including new MDMA-related clinical cases. Eur Neuropsychopharmacol (2014) 24(8):1309–23. 10.1016/j.euroneuro.2014.05.008 24933532

[217] Buckholtz NS Zhou DF Freedman DX. Serotonin2 agonist administration down-regulates rat brain serotonin2 receptors. Life Sci (1988) 42(24):2439–45. 10.1016/0024-3205(88)90342-6 3374263

[218] Buckholtz NS Zhou DF Freedman DX Potter WZ. Lysergic acid diethylamide (LSD) administration selectively downregulates serotonin2 receptors in rat brain. Neuropsychopharmacology (1990) 3(2):137–48. 1969270

[219] Roth BL Willins DL Kroeze WK G protein-coupled receptor (GPCR) trafficking in the central nervous system: relevance for drugs of abuse. Drug Alcohol Depend (1998) 51(1–2):73–85. 10.1016/S0376-8716(98)00067-2 9716931

[220] Aloyo VJ Dave KD Rahman T Harvey JA. Selective and divergent regulation of cortical 5-HT(2A) receptors in rabbit. J Pharmacol Exp Ther (2001) 299(3):1066–72. 11714896

[221] Gresch PJ Smith RL Barrett RJ Sanders-Bush E. Behavioral tolerance to lysergic acid diethylamide is associated with reduced serotonin-2A receptor signaling in rat cortex. Neuropsychopharmacology (2005) 30(9):1693–702. 10.1038/sj.npp.1300711 15756304

[222] Buchborn T Schroder H Dieterich DC Grecksch G Hollt V. Tolerance to LSD and DOB induced shaking behaviour: differential adaptations of frontocortical 5-HT(2A) and glutamate receptor binding sites. Behav Brain Res (2015) 281:62–8. 10.1016/j.bbr.2014.12.014 25513973

[223] Capela JP Fernandes E Remiao F Bastos ML Meisel A Carvalho F. Ecstasy induces apoptosis via 5-HT(2A)-receptor stimulation in cortical neurons. Neurotoxicology (2007) 28(4):868–75. 10.1016/j.neuro.2007.04.005 17572501

[224] Capela JP da Costa Araujo S Costa VM Ruscher K Fernandes E Bastos Mde L The neurotoxicity of hallucinogenic amphetamines in primary cultures of hippocampal neurons. Neurotoxicology (2013) 34:254–63. 10.1016/j.neuro.2012.09.005 22983118

[225] Capela JP Ruscher K Lautenschlager M Freyer D Dirnagl U Gaio AR Ecstasy-induced cell death in cortical neuronal cultures is serotonin 2A-receptor-dependent and potentiated under hyperthermia. Neuroscience (2006) 139(3):1069–81. 10.1016/j.neuroscience.2006.01.007 16504407

[226] Noworyta-Sokolowska K Kaminska K Kreiner G Rogoz Z Golembiowska K. Neurotoxic effects of 5-MeO-DIPT: a psychoactive tryptamine derivative in rats. Neurotox Res (2016) 30(4):606–19. 10.1007/s12640-016-9654-0 27461536 PMC5047954

[227] King W Jr Ellison G. Long-lasting alterations in behavior and brain neurochemistry following continuous low-level LSD administration. Pharmacol Biochem Behav (1989) 33(1):69–73. 10.1016/0091-3057(89)90431-0 2780790

[228] Karila L Reynaud M. GHB and synthetic cathinones: clinical effects and potential consequences. Drug Test Anal (2011) 3:552–9. 10.1002/dta.210 21960540

[229] Sanchez S Sur un homologue de l’ephedrine. Bull Soc Chim Fr (1929) 45:284–6.

[230] Green AR King MV Shortall SE Fone KC. The preclinical pharmacology of mephedrone; not just MDMA by another name. Br J Pharmacol (2014) 171:2251–68. 10.1111/bph.12628 24654568 PMC3997268

[231] Karila L Billieux J Benyamina A Lancon C Cottencin O. The effects and risks associated to mephedrone and methylone in humans: a review of the preliminary evidences. Brain Res Bull (2016) 126:61–7. 10.1016/j.brainresbull.2016.03.005 26995278

[232] Brunt TM Poortman A Niesink RJ van den Brink W. Instability of the ecstasy market and a new kid on the block: mephedrone. J Psychopharmacol (2011) 25:1543–7. 10.1177/0269881110378370 20826554

[233] Cottencin O Rolland B Karila L. New designer drugs (synthetic cannabinoids and synthetic cathinones): review of literature. Curr Pharm Des (2014) 20:4106–11. 10.2174/13816128113199990622 24001292

[234] EMCDDA. EMCDDA and Europol Step Up Information Collection on Mephedrone. Lisbon: Drugnet Europe (2010).

[235] EMCDDA. Risk Assessment Report of a New Psychoactive Substance: 4-Methylmethcathinone (Mephedrone). In Accordance with Article 6 of Council Decision 2005/387/JHA on Information Exchange, Risk Assessment and Control of New Psychoactive Substances. Lisbon: European Monitoring Centre for Drugs and Drug Addiction (EMCDDA) (2010).

[236] EMCDDA. Annual Report 2011: The State of the Drug Problem in Europe. Lisbon: European Monitoring Center for Drugs and Drug Abuse (2012).

[237] Debruyne D Courné MA Le Boisselier R Djezzar S Gérardin M Boucher A Mephedrone: a designer drug of recent use in France. Therapie (2010) 65:519–24. 10.2515/therapie/2010077 21176758

[238] Vardakou I Pistos C Spiliopoulou C. Drugs for youth via Internet and the example of mephedrone. Toxicol Lett (2011) 201:191–5. 10.1016/j.toxlet.2010.12.014 21187132

[239] Martinotti G Lupi M Carlucci L Cinosi E Santacroce R Acciavatti T Novel psychoactive substances: use and knowledge among adolescents and young adults in urban and rural areas. Hum Psychopharmacol (2015) 30:295–301. 10.1002/hup.2486 26216566

[240] Zawilska JB Wojcieszak J Designer cathinones—an emerging class of novel recreational drugs. Forensic Sci Int (2013) 231:42–53. 10.1016/j.forsciint.2013.04.015 23890615

[241] Glennon RA. Bath salts mephedrone, and methylenedioxypyrovalerone as emerging illicit drugs that will need targeted therapeutic intervention. Adv Pharmacol (2014) 69:581–620. 10.1016/B978-0-12-420118-7.00015-9 24484988 PMC4471862

[242] Schifano F Albanese A Fergus S Stair JL Deluca P Corazza O Mephedrone (4-methylmethcathinone; ’meow meow’): chemical, pharmacological and clinical issues. Psychopharmacology (2011) 214:593–602. 10.1007/s00213-010-2070-x 21072502

[243] Kelly JP. Cathinone derivatives: a review of their chemistry, pharmacology and toxicology. Drug Test Anal (2011) 3:439–53. 10.1002/dta.313 21755607

[244] Karila L Megarbane B Cottencin O Lejoyeux M. Synthetic cathinones: a new public health problem. Curr Neuropharmacol (2015) 13:12–20. 10.2174/1570159X13666141210224137 26074740 PMC4462036

[245] Wood DM Dargan PI. Mephedrone (4-methylmethcathinone): what is new in our understanding of its use and toxicity. Prog Neuropsychopharmacol Biol Psychiatry (2012) 39:227–33. 10.1016/j.pnpbp.2012.04.020 22564711

[246] German CL Fleckenstein AE Hanson GR. Bath salts and synthetic cathinones: an emerging designer drug phenomenon. Life Sci (2014) 97:2–8. 10.1016/j.lfs.2013.07.023 23911668 PMC3909723

[247] Kapitány-Fövény M Mervó B Kertész M Corazza O Farkas J Kökönyei G Is there any difference in patterns of use and psychiatric symptom status between injectors and non-injectors of mephedrone? Hum Psychopharmacol (2015) 30:233–43. 10.1002/hup.2490 26216556

[248] Van Hout MC Bingham T A costly turn on: patterns of use and perceived consequences of mephedrone based head shop products amongst Irish injectors. Int J Drug Policy (2012) 23:188–97. 10.1016/j.drugpo.2012.01.008 22342322

[249] Wood DM Greene SL Dargan PI. Clinical pattern of toxicity associated with the novel synthetic cathinone mephedrone. Emerg Med J (2011) 28:280–2. 10.1136/emj.2010.092288 20581379

[250] John ME Thomas-Rozea C Hahn D Bath salts abuse leading to new onset psychosis and potential for violence. Clin Schizophr Relat Psychoses (2017) 11(2):120–4. 10.3371/CSRP.JORO.061314 24951715

[251] Dragogna F Oldani L Buoli M Altamura AC. A case of severe psychosis induced by novel recreational drugs. F1000Res (2014) 3:21. 10.12688/f1000research.3-21.v1 25352977 PMC4207243

[252] Bajaj N Mullen D Wylie S. Dependence and psychosis with 4-methylmethcathinone (mephedrone) use. BMJ Case Rep (2010) 2010:1–3. 10.1136/bcr.02.2010.2780 22791836 PMC3027483

[253] Loi B Corkery JM Claridge H Goodair C Chiappini S Gimeno Clemente C Deaths of individuals aged 16–24 years in the UK after using mephedrone. Hum Psychopharmacol (2015) 30:225–32. 10.1002/hup.2423 26216555

[254] Gustavsson D Escher C Mephedrone—Internet drug which seems to have come and stay. Fatal cases in Sweden have drawn attention to previously unknown substance. Lakartidningen (2009) 106(43):2769–71. 19960905

[255] Schifano F Corkery J Ghodse A Suspected and confirmed fatalities associated with mephedrone (4-methylmethcathinone, Meow Meow) in the United Kingdom. J Clin Psychopharmacol (2012) 32:710–4. 10.1097/JCP.0b013e318266c70c 22926609

[256] Busardò FP Kyriakou C Napoletano S Marinelli E Zaami S. Mephedrone related fatalities: a review. Eur Rev Med Pharmacol Sci (2015) 19:3777–90. 26502870

[257] Prosser JM Nelson LS. The toxicology of bath salts: a review of synthetic cathinones. J Med Toxicol (2012) 8:33–42. 10.1007/s13181-011-0193-z 22108839 PMC3550219

[258] Morris H Mephedrone: The Phantom Menace. Montreal: Vice (2010). p. 98–100.

[259] Winder GS Stern N Hosanagar A Are bath salts the next generation of stimulant abuse? J Subst Abuse Treat (2013) 44:42–5. 10.1016/j.jsat.2012.02.003 22445773

[260] Carhart-Harris RL King LA Nutt DJ. A web-based survey on mephedrone. Drug Alcohol Depend (2011) 118:19–22. 10.1016/j.drugalcdep.2011.02.011 21420252

[261] Winstock AR Mitcheson LR Deluca P Davey Z Corazza O Schifano F. Mephedrone, new kid for the chop? Addiction (2011) 106:154–61. 10.1111/j.1360-0443.2010.03130.x 20735367

[262] Simmler LD Buser TA Donzelli M Schramm Y Dieu LH Huwyler J Pharmacological characterization of designer cathinones in vitro. Br J Pharmacol (2013) 168:458–70. 10.1111/j.1476-5381.2012.02145.x 22897747 PMC3572571

[263] Meyer MR Maurer HH. Metabolism of designer drugs of abuse: an updated review. Curr Drug Metab (2010) 11:468–82. 10.2174/138920010791526042 20540700

[264] Jacob P Shulgin A Inventors; Neurobiological Technologies, Inc., assignee Preparation of Novel N-Substituted-2-Amino-3,4-Methylenedioxypropiophenones As Anti-Depressant and Anti-Parkinsonism Agents. (1996). US Patent WO9639133.

[265] Meyer MR Wilhelm J Peters FT Maurer HH Beta-keto amphetamines: studies on the metabolism of the designer drug mephedrone and toxicological detection of mephedrone, butylone, and methylone in urine using gas chromatography-mass spectrometry. Anal Bioanal Chem (2010) 397:1225–33. 10.1007/s00216-010-3636-5 20333362

[266] EMCDDA. EMCDDA–Europol 2009 Annual Report on the Implementation of Council Decision 2005/387/JHA. In Accordance with Article 10 of Council Decision 2005/387/JHA on the Information Exchange, Risk-Assessment and Control of New Psychoactive Substances. Lisbon: EMCDDA–Europol joint publication (2010).

[267] McNamara S Stokes S Coleman N. Head shop compound abuse amongst attendees of the Drug Treatment Centre Board. Ir Med J (2010) 103:134–7. 20666082

[268] WHO. Methylone (bk-MDMA): critical review report. Expert Committee on Drug Dependence Thirty-Sixth Meeting Geneva (2014).

[269] De Felice LJ Glennon RA Negus SS. Synthetic cathinones: chemical phylogeny physiology, and neuropharmacology. Life Sci (2014) 97:20–6. 10.1016/j.lfs.2013.10.029 24231923 PMC3944897

[270] Shimizu E Watanabe H Kojima T Hagiwara H Fujisaki M Miyatake R Combined intoxication with methylone and 5-MeO-MIPT. Prog Neuropsychopharmacol Biol Psychiatry (2007) 31:288–91. 10.1016/j.pnpbp.2006.06.012 16876302

[271] Boulanger-Gobeil C St-Onge M Laliberté M Auger PL. Seizures and hyponatremia related to ethcathinone and methylone poisoning. J Med Toxicol (2012) 8:59–61. 10.1007/s13181-011-0159-1 21755421 PMC3550214

[272] Piao YS Hall FS Moriya Y Ito M Ohara A Kikura-Hanajiri R Methylone-induced hyperthermia and lethal toxicity: role of the dopamine and serotonin transporters. Behav Pharmacol (2015) 26:345–52. 10.1097/FBP.0000000000000135 25794333

[273] Pearson JM Hargraves TL Hair LS Massucci CJ Frazee CC III Garg U Three fatal intoxications due to methylone. J Anal Toxicol (2012) 36:444–51. 10.1093/jat/bks043 22589523

[274] Warrick BJ Wilson J Hedge M Freeman S Leonard K Aaron C. Lethal serotonin syndrome after methylone and butylone ingestion. J Med Toxicol (2012) 8:65–8. 10.1007/s13181-011-0199-6 22160789 PMC3550225

[275] Carbone PN Carbone DL Carstairs SD Luzi SA. Sudden cardiac death associated with methylone use. Am J Forensic Med Pathol (2013) 34:26–8. 10.1097/PAF.0b013e31827ab5da 23403480

[276] Barrios L Grison-Hernando H Boels D Bouquie R Monteil-Ganiere C Clement R Death following ingestion of methylone. Int J Legal Med (2015) 130:381–5. 10.1007/s00414-015-1212-4 26071183

[277] Bossong MG Van Dijk JP Niesink RJ. Methylone and mCPP, two new drugs of abuse? Addict Biol (2005) 10:321–3. 10.1080/13556210500350794 16318952

[278] Koob GF Volkow ND. Neurobiology of addiction: a neurocircuitry analysis. Lancet Psychiatry (2016) 3:760–73. 10.1016/S2215-0366(16)00104-8 27475769 PMC6135092

[279] Müller CP Homberg JR. The role of serotonin in drug use and addiction. Behav Brain Res (2015) 277:146–92. 10.1016/j.bbr.2014.04.007 24769172

[280] Morris H Wallach J. From PCP to MXE: a comprehensive review of the non-medical use of dissociative drugs. Drug Test Anal (2014) 6(7–8):614–32. 10.1002/dta.1620 24678061

[281] Breen C Degenhardt L White B Bruno R Chanteloup F Fisher J Australian Party Drugs Trends 2003: Findings from the Party Drug Initiative. Sydney: National Drug and Alcohol Research Centre (2008).

[282] Kurdi MS Theerth KA Deva RS. Ketamine: current applications in anesthesia, pain, and critical care. Anesth Essays Res (2014) 8:283–90. 10.4103/0259-1162.143110 25886322 PMC4258981

[283] Coyle CM Laws KR The use of ketamine as an antidepressant: a systematic review and meta-analysis. Hum Psychopharmacol Clin Exp (2015) 30:233–7. 10.1002/hup.2475 25847818

[284] Rowland LM. Subanesthetic ketamine: how it alters physiology and behavior in humans. Aviat Space Environ Med (2005) 76(7, Suppl.):C52–8. 16018330

[285] Sheehy KA Muller EA Lippold C Nouraie M Finkel JC Quezado ZM. Subanesthetic ketamine infusions for the treatment of children and adolescents with chronic pain: a longitudinal study. BMC Pediatr (2015) 15:198. 10.1186/s12887-015-0515-4 26620833 PMC4665913

[286] Gable RS. Acute toxic effects of club drugs. J Psychoactive Drugs (2004) 36:303–13. 10.1080/02791072.2004.10400031 15559678

[287] Morgan CJ Curran HV Independent Scientific Committee on Drugs Ketamine use: a review. Addiction (2012) 107:27–38. 10.1111/j.1360-0443.2011.03576.x 21777321

[288] Bokor G Anderson PD. Ketamine: an update on its abuse. J Pharm Pract (2014) 27:582–6. 10.1177/0897190014525754 24651639

[289] Reynaud-Maurupt C Bello PY Akoka S Toufik A. Characteristics and behaviors of ketamine users in France in 2003. J Psychoactive Drugs (2007) 39:1–11. 10.1080/02791072.2007.10399859 17523580

[290] Britt GC McCance-Katz EF A brief overview of the clinical pharmacology of club drugs. Subst Use Misuse (2005) 40:1189–201. 10.1081/JA-200066730 16048813

[291] Müller CP Pum ME Amato D Schüttler J Huston JP de Souza Silva MA The in-vivo neurochemistry of the brain during general anesthesia. J Neurochem (2011) 119:419–46. 10.1111/j.1471-4159.2011.07445.x 21883214

[292] Powers AR III Gancsos MG Finn ES Morgan PT Corlett PR. Ketamine-induced hallucinations. Psychopathology (2015) 48:376–85. 10.1159/000438675 26361209 PMC4684980

[293] Zhuo X Cang Y Yan H Bu J Shen B. The prevalence of drugs in motor vehicle accidents and traffic violations in Shanghai and neighboring cities. Accid Anal Prev (2010) 42:2179–84. 10.1016/j.aap.2010.07.004 20728679

[294] Giorgetti R Marcotulli D Tagliabracci A Schifano F. Effects of ketamine on psychomotor, sensory and cognitive functions relevant for driving ability. Forensic Sci Int (2015) 252:127–42. 10.1016/j.forsciint.2015.04.024 25981945

[295] Penning R Veldstra JL Daamen AP Olivier B Verster JC. Drugs of abuse, driving and traffic safety. Curr Drug Abuse Rev (2010) 3:23–32. 10.2174/1874473711003010023 20088818

[296] Pappas MK Halkitis PN. Sexual risk taking and club drug use across three age cohorts of HIV-positive gay and bisexual men in New York City. AIDS Care (2011) 23:1410–6. 10.1080/09540121.2011.565027 22022849 PMC3205430

[297] Xu JJ Zhang C Hu QH Chu ZX Zhang J Li YZ Recreational drug use and risks of HIV and sexually transmitted infections among Chinese men who have sex with men: mediation through multiple sexual partnerships. BMC Infect Dis (2014) 14:642. 10.1186/s12879-014-0642-9 25443542 PMC4272794

[298] Weiner A Vieira L McKay CA Bayer MJ. Ketamine abusers presenting to the emergency department: a case series. J Emerg Med (2000) 18:447–51. 10.1016/S0736-4679(00)00162-1 10802423

[299] Mason K Cottrell AM Corrigan AG Gillatt DA Mitchelmore AE. Ketamine-associated lower urinary tract destruction: a new radiological challenge. Clin Radiol (2010) 65:795–800. 10.1016/j.crad.2010.05.003 20797465

[300] Skeldon SC Goldenberg SL. Urological complications of illicit drug use. Nat Rev Urol (2014) 11:169–77. 10.1038/nrurol.2014.22 24535583

[301] Yek J Sundaram P Aydin H Kuo T Ng LG. The clinical presentation and diagnosis of ketamine-associated urinary tract dysfunction in Singapore. Singapore Med J (2015) 56:660–5. 10.11622/smedj.2015185 26702160 PMC4678404

[302] Huang LK Wang JH Shen SH Lin AT Chang CY Evaluation of the extent of ketamine-induced uropathy: the role of CT urography. Postgrad Med J (2014) 90:185–90. 24443558 10.1136/postgradmedj-2013-131776 PMC3963547

[303] Wu S Lai Y He Y Li X Guan Z Cai Z. Lower urinary tract destruction due to ketamine: a report of 4 cases and review of literature. J Addict Med (2012) 6:85–8. 10.1097/ADM.0b013e3182312863 21979820

[304] Chen WY Huang MC Lin SK. Gender differences in subjective discontinuation symptoms associated with ketamine use. Subst Abuse Treat Prev Policy (2014) 9:39. 10.1186/1747-597X-9-39 25245125 PMC4183767

[305] Tang J Liao Y He H Deng Q Zhang G Qi C Sleeping problems in Chinese illicit drug dependent subjects. BMC Psychiatry (2015) 15:28. 10.1186/s12888-015-0409-x 25884573 PMC4337091

[306] Costi S Dam NTV Murrough JW. Current status of ketamine and related therapies for mood and anxiety disorders. Curr Behav Neurosci Rep (2015) 2:216–25. 10.1007/s40473-015-0052-3 26783510 PMC4714563

[307] Bonnet U. Long-term ketamine self-injections in major depressive disorder: focus on tolerance in ketamine’s antidepressant response and the development of ketamine addiction. J Psychoactive Drugs (2015) 47(4):276–85. 10.1080/02791072.2015.1072653 26317449

[308] Pender JW Dissociative anesthesia. Calif Med (1970) 113(5):73. PMC1501800 18730444

[309] Petersen RC Stillman RC Phencyclidine: an overview. NIDA Res Monogr (1978) 21:1–17. 101864

[310] Morgan CJ Curran HV. Acute and chronic effects of ketamine upon human memory: a review. Psychopharmacology (Berl) (2006) 188(4):408–24. 10.1007/s00213-006-0572-3 17006715

[311] Rocha A Hart N Trujillo KA. Differences between adolescents and adults in the acute effects of PCP and ketamine and in sensitization following intermittent administration. Pharmacol Biochem Behav (2017) 157:24–34. 10.1016/j.pbb.2017.04.007 28442368 PMC5540233

[312] Liang HJ Tang KL Chan F Ungvari GS Tang WK. Ketamine users have high rates of psychosis and/or depression. J Addict Nurs (2015) 26(1):8–13. 10.1097/JAN.0000000000000060 25761158

[313] Stone JM Pepper F Fam J Furby H Hughes E Morgan C Glutamate, N-acetyl aspartate and psychotic symptoms in chronic ketamine users. Psychopharmacology (Berl) (2014) 231(10):2107–16. 10.1007/s00213-013-3354-8 24264567

[314] Kamal RM Van Noorden MS Wannet W Beurmajer H Dijkstra BA Schellekens A Pharmacological treatment in gamma-hydroxybutyrate (GHB) and gamma-butyrolactone (GBL) dependence: detoxification and relapse prevention. CNS Drugs (2017) 31:51–64. 10.1007/s40263-016-0402-z 28004314

[315] Carai MA Colombo G Reali R Serra S Mocci I Castelli MP Central effects of 1,4-butanediol are mediated by GABA(B) receptors via its conversion into gamma-hydroxybutyric acid. Eur J Pharmacol (2002) 441:157–63. 10.1016/S0014-2999(02)01502-9 12063087

[316] Irwing RD NTP Summary Report on the Metabolism, Disposition and Toxicity of 1,4-Butanediol. North Carolina: USDoHaH Services (1996). 11803699

[317] Bessman SP Fishbein WN Gamma-hydroxybutyrate, a normal brain metabolite. Nature (1963) 200:1207–8. 10.1038/2001207a0 14089913

[318] Snead OC III Morley BJ. Ontogeny of gamma-hydroxybutyric acid. I. Regional concentration in developing rat, monkey and human brain. Brain Res (1981) 2227:579–89. 10.1016/0165-3806(81)90010-9 7260659

[319] Engberg G Nissbrandt H. Gamma-hydroxybutyric acid (GHBA) induces pacemaker activity and inhibition of substantia nigra dopamine neurons by activating GABAB-receptors. Naunyn Schmiedebergs Arch Pharmacol (1993) 348:491–7. 10.1007/BF00173208 8114948

[320] Benavides J Rumigny JF Bourguignon JJ Cash C Wermuth CG Mandel P High affinity binding sites for gamma-hydroxybutyric acid in rat brain. Life Sci (1982) 30:953–61. 10.1016/0024-3205(82)90624-5 7070203

[321] Snead OC III. Evidence for a G protein-coupled gamma-hydroxybutyric acid receptor. J Neurochem (2000) 75:1986–96. 10.1046/j.1471-4159.2000.0751986.x 11032888

[322] Carter LP Koek W France CP Behavioral analyses of GHB: receptor mechanisms. Pharmacol Ther (2009) 121:100–14. 10.1016/j.pharmthera.2008.10.003 19010351 PMC2631377

[323] Andresen H Aydin BE Mueller A Iwersen-Bergmann S. An overview of gamma-hydroxybutyric acid: pharmacodynamics, pharmacokinetics, toxic effects, addiction, analytical methods, and interpretation of results. Drug Test Anal (2011) 3:560–8. 10.1002/dta.254 21381220

[324] Liechti ME Quednow BB Liakoni E Dornbierer D von Rotz R Gachet MS Pharmacokinetics and pharmacodynamics of gamma-hydroxybutyrate in healthy subjects. Br J Clin Pharmacol (2016) 81(5):980–8. 10.1111/bcp.12863 26659543 PMC4834605

[325] Schep LJ Knudsen K Slaughter RJ Vale JA Mégarbane B The clinical toxicology of gamma-hydroxybutyrate, gamma-butyrolactone and 1,4-butanediol. Clin Toxicol (Phila) (2012) 50:458–70. 10.3109/15563650.2012.702218 22746383

[326] Kaufman EE Nelson T Miller D Stadlan N. Oxidation of gamma-hydroxybutyrate to succinic semialdehyde by a mitochondrial pyridine nucleotide-independent enzyme. J Neurochem (1988) 51:1079–84. 10.1111/j.1471-4159.1988.tb03071.x 3138385

[327] Abanades S Farre M Segura M Pichini S Barral D Pacifici R Gamma-hydroxybutyrate (GHB) in humans: pharmacodynamics and pharmacokinetics. Ann N Y Acad Sci (2006) 1074:559–76. 10.1196/annals.1369.065 17105953

[328] Bosch OG Eisenegger C Gertsch J von Rotz R Dornbierer D Gachet MS Gamma-hydroxybutyrate enhances mood and prosocial behavior without affecting plasma oxytocin and testosterone. Psychoneuroendocrinology (2015) 62:1–10. 10.1016/j.psyneuen.2015.07.167 26209926

[329] Miotto K Darakjian J Basch J Murray S Zogg J Rawson R. Gamma-hydroxybutyric acid: patterns of use, effects and withdrawal. Am J Addict (2001) 10:232–41. 10.1080/105504901750532111 11579621

[330] Van Sassenbroeck DK De Neve N De Paepe P Belpaire FM Verstraete AG Calle PA Abrupt awakening phenomenon associated with gamma-hydroxybutyrate use: a case series. Clin Toxicol (Phila) (2007) 45:533–8. 10.1080/15563650701365818 17503262

[331] Donjacour CE Aziz NA Overeem S Kalsbeek A Pijl H Lammers GJ. Glucose and fat metabolism in narcolepsy and the effect of sodium oxybate: a hyperinsulinemic-euglycemic clamp study. Sleep (2014) 37:795–801. 10.5665/sleep.3592 24899766 PMC4044740

[332] Goodwin AK Froestl W Weerts EM. Involvement of gamma-hydroxybutyrate (GHB) and GABA-B receptors in the acute behavioral effects of GHB in baboons. Psychopharmacology (Berl) (2005) 180:342–51. 10.1007/s00213-005-2165-y 15739078

[333] Husain AM Ristanovic RK Bogan RK. Weight loss in narcolepsy patients treated with sodium oxybate. Sleep Med (2009) 10:661–3. 10.1016/j.sleep.2008.05.012 19014899

[334] Ottani A Leone S Vergara FB Tacchi R Loche A Bertolini A. Preference for palatable food is reduced by the gamma-hydroxybutyrate analogue GET73, in rats. Pharmacol Res (2007) 55:271–9. 10.1016/j.phrs.2006.12.002 17240159

[335] McElroy SL Guerdjikova AI Winstanley EL O’Melia AM Mori N Keck PE Jr Sodium oxybate in the treatment of binge eating disorder: an open-label, prospective study. Int J Eat Disord (2011) 44:262–8. 10.1002/eat.20798 20209489

[336] Kapitány-Fövény M Mervó B Corazza O Kökönyei G Farkas J Urbán R Enhancing sexual desire and experience: an investigation of the sexual correlates of gamma-hydroxybutyrate (GHB) use. Hum Psychopharmacol (2015) 30:276–84. 10.1002/hup.2491 26216563

[337] Sumnall HR Woolfall K Edwards S Cole JC Beynon CM. Use, function, and subjective experiences of gamma-hydroxybutyrate (GHB). Drug Alcohol Depend (2008) 92:286–90. 10.1016/j.drugalcdep.2007.07.009 17766059

[338] Teltzrow R Bosch OG Ecstatic anaesthesia: ketamine and GHB between medical use and self-experimentation. Appl Cardiopulm Pathophysiol (2012) 16:309–21.

[339] Bosch OG Havranek MM Baumberger A Preller KH von Rotz R Herdener M Neural underpinnings of prosexual effects induced by gamma-hydroxybutyrate in healthy male humans. Eur Neuropsychopharmacol (2017) 27(4):372–82. 10.1016/j.euroneuro.2017.02.006 28284776

[340] Mamelak M Escriu JM Stokan O Sleep-inducing effects of gammahydroxybutyrate. Lancet (1973) 2:328–9. 10.1016/S0140-6736(73)90839-8 4124818

[341] Black J Houghton WC. Sodium oxybate improves excessive daytime sleepiness in narcolepsy. Sleep (2006) 29:939–46. 10.1093/sleep/29.7.939 16895262

[342] Black J Pardi D Hornfeldt CS Inhaber N. The nightly use of sodium oxybate is associated with a reduction in nocturnal sleep disruption: a double-blind, placebo-controlled study in patients with narcolepsy. J Clin Sleep Med (2010) 6(6):596–602. 21206549 PMC3014247

[343] Spaeth M Bennett RM Benson BA Wang YG Lai C Choy EH. Sodium oxybate therapy provides multidimensional improvement in fibromyalgia: results of an international phase 3 trial. Ann Rheum Dis (2012) 71:935–42. 10.1136/annrheumdis-2011-200418 22294641 PMC3371223

[344] Jimeńez F Lopez Timoneda F Nodal C Arrigain S Neurophysiological study of the effects of gamma-OH at several doses in normal subjects. Arch Neurobiol (Madr) (1982) 45:3–28. 7125804

[345] Metcalf DR Emde RN Stripe JT An EEG-behavioral study of sodium hydroxybutyrate in humans. Electroencephalogr Clin Neurophysiol (1966) 20:506–12. 10.1016/0013-4694(66)90107-6 4143689

[346] Van Cauter E Plat L Scharf MB Leproult R Cespedes S L’Hermite-Balériaux M Simultaneous stimulation of slow-wave sleep and growth hormone secretion by gamma-hydroxybutyrate in normal young men. J Clin Invest (1997) 100:745–53. 10.1172/JCI119587 9239423 PMC508244

[347] von Rotz R Kometer M Dornbierer D Gertsch J Salomé Gachet M Vollenweider FX Neuronal oscillations and synchronicity associated with gamma-hydroxybutyrate during resting-state in healthy male volunteers. Psychopharmacology (Berl) (2017) 234:1957–68. 10.1007/s00213-017-4603-z 28429067

[348] Bosch OG Esposito F Havranek MM Dornbierer D von Rotz R Staempfli P Gamma-hydroxybutyrate increases resting state limbic perfusion and body and emotion awareness in humans. Neuropsychopharmacology. (2017) 10.1038/npp.2017.110. PMC5603804 28561068

[349] EMCDDA. GHB and Its Precursor GBL: An Emerging Trend Case Study. Lisbon: European Monitoring Centre for Drugs and Drug Addiction (EMCDDA) (2008).

[350] WHO. Gamma-hydroxybutyric acid (GHB) critical review report. WHO Expert Committee on Drug DependeNCE, ECOD – Thirty-fifth Meeting Hammamet, Tunisia (2012).

[351] Carter LP Pardi D Gorsline J Griffiths RR Illicit gamma-hydroxybutyrate (GHB) and pharmaceutical sodium oxybate (Xyrem): differences in characteristics and misuse. Drug Alcohol Depend (2009) 104:1–10. 10.1016/j.drugalcdep.2009.04.012 19493637 PMC2713368

[352] USGPO. Public Law 106–172 – Hillory J. Farias and Samantha Reid Date-Rape Drug Prohibition Act of 2000. U.S. Government Publishing Office (2000). Available from: http://www.gpo.gov/fdsys/pkg/PLAW-106publ172/content-detail.html

[353] Nemeth Z Kun B Demetrovics Z. The involvement of gamma-hydroxybutyrate in reported sexual assaults: a systematic review. J Psychopharmacol (2010) 24:1281–7. 10.1177/0269881110363315 20488831

[354] Boscolo-Berto R Viel G Montagnese S Raduazzo DI Ferrara SD Dauvilliers Y. Narcolepsy and effectiveness of gamma-hydroxybutyrate (GHB): a systematic review and meta-analysis of randomized controlled trials. Sleep Med Rev (2012) 16:431–43. 10.1016/j.smrv.2011.09.001 22055895

[355] Caputo F Mirijello A Cibin M Mosti A Ceccanti M Domenicali M Novel strategies to treat alcohol dependence with sodium oxybate according to clinical practice. Eur Rev Med Pharmacol Sci (2015) 19:1315–20. 25912595

[356] Russell IJ Perkins AT Michalek JE Oxybate SXB-26 Fibromyalgia Syndrome Study Group. Sodium oxybate relieves pain and improves function in fibromyalgia syndrome: a randomized, double-blind, placebo-controlled, multicenter clinical trial. Arthritis Rheum (2009) 60:299–309. 10.1002/art.24142 19116896

[357] Russell IJ Holman AJ Swick TJ Alvarez-Horine S Wang YG Guinta D Sodium oxybate reduces pain, fatigue, and sleep disturbance and improves functionality in fibromyalgia: results from a 14-week, randomized, double-blind, placebo-controlled study. Pain (2011) 152:1007–17. 10.1016/j.pain.2010.12.022 21397402

[358] Spaeth M Alegre C Perrot S Wang Y Guinta DR Alvarez-Horine S Long-term tolerability and maintenance of therapeutic response to sodium oxybate in an open-label extension study in patients with fibromyalgia. Arthritis Res Ther (2013) 15:R185. 10.1186/ar4375 24286114 PMC3978755

[359] Kantrowitz JT Citrome L Javitt DC A review of tolerability and abuse liability of gamma-hydroxybutyric acid for insomnia in patients with schizophrenia. Clin Ther (2009) 31(Pt 1):1360–73. 10.1016/j.clinthera.2009.07.005 19698899

[360] Ondo WG Perkins T Swick T Hull KL Jr Jimenez JE Garris TS Sodium oxybate for excessive daytime sleepiness in Parkinson disease: an open-label polysomnographic study. Arch Neurol (2008) 65:1337–40. 10.1001/archneur.65.10.1337 18852348

[361] Hidalgo H Uhl V Gantenbein AR Sándor PS Kallweit U. Efficiency of sodium oxybate in episodic cluster headache. Headache (2013) 53:1490–1. 10.1111/head.12068 23463909

[362] Khatami R Tartarotti S Siccoli MM Bassetti CL Sándor PS. Long-term efficacy of sodium oxybate in 4 patients with chronic cluster headache. Neurology (2011) 77:67–70. 10.1212/WNL.0b013e31822313c6 21613599

[363] Bosch OG Quednow BB Seifritz E Wetter TC. Reconsidering GHB: orphan drug or new model antidepressant? J Psychopharmacol (2012) 26:618–28. 10.1177/0269881111421975 21926421

[364] Mamelak M. Narcolepsy and depression and the neurobiology of gammahydroxybutyrate. Prog Neurobiol (2009) 89:193–219. 10.1016/j.pneurobio.2009.07.004 19654034

[365] Carter LP Richards BD Mintzer MZ Griffiths RR. Relative abuse liability of GHB in humans: a comparison of psychomotor, subjective, and cognitive effects of supratherapeutic doses of triazolam, pentobarbital, and GHB. Neuropsychopharmacology (2006) 31:2537–51. 10.1038/sj.npp.1301146 16880774

[366] Chin MY Kreutzer RA Dyer JE. Acute poisoning from gamma-hydroxybutyrate in California. West J Med (1992) 156:380–4. 1574880 PMC1003276

[367] George CF Feldman N Inhaber N Steininger TL Grzeschik SM Lai C A safety trial of sodium oxybate in patients with obstructive sleep apnea: acute effects on sleep-disordered breathing. Sleep Med (2010) 11:38–42. 10.1016/j.sleep.2009.06.006 19897413

[368] Bosch OG Seifritz E The behavioural profile of gamma-hydroxybutyrate, y-butyrolactone and 1,4-butanediol in humans. Brain Res Bull (2016) 126(Pt 1):47–60. 10.1016/j.brainresbull.2016.02.002 26855327

